# The potential global cost-effectiveness of prospective Strep A vaccines and associated implementation efforts

**DOI:** 10.1038/s41541-023-00718-7

**Published:** 2023-08-25

**Authors:** Jung-Seok Lee, Vittal Mogasale, Sol Kim, Jeffrey Cannon, Fiona Giannini, Kaja Abbas, Jean-Louis Excler, Jerome H. Kim

**Affiliations:** 1 International Vaccine Institute, Seoul, South Korea; 2 Telethon Kids Institute, Perth, Australia; 3 London School of Hygiene & Tropical Medicine, London, United Kingdom; 4 College of Natural Sciences, Seoul National University, Seoul, South Korea

**Keywords:** Epidemiology, Translational research

## Abstract

Group A Streptococcus causes a wide range of diseases from relatively mild infections including pharyngitis to more severe illnesses such as invasive diseases and rheumatic heart disease (RHD). Our aim is to estimate the cost-effectiveness of a hypothetical Strep A vaccine on multiple disease manifestations at the global-level. Cost-effectiveness analyses were carried out by building on the potential epidemiological impact of vaccines that align with the WHO’s Preferred Product Characteristics for Strep A vaccines. Maximum vaccination costs for a cost-effective vaccination strategy were estimated at the thresholds of 1XGDP per capita and health opportunity costs. The maximum cost per fully vaccinated person for Strep A vaccination to be cost-effective was $385–$489 in high-income countries, $213–$312 in upper-income-income countries, $74–$132 in lower-middle-income countries, and $37–$69 in low-income countries for routine vaccination at birth and 5 years of age respectively. While the threshold costs are sensitive to vaccine characteristics such as efficacy, and waning immunity, a cost-effective Strep A vaccine will lower morbidity and mortality burden in all income settings.

## Introduction

Group A Streptococcus (Strep A) is one of the leading causes of global death and disability, particularly posing a greater threat in resource-limited countries^1^. Strep A causes a wide range of clinical manifestations from seemingly benign superficial infection to severe invasive diseases, as well as chronic and lethal rheumatic heart disease (RHD)^1,2^. While an accurate, contemporary estimate of the global disease burden of Strep A remains limited due to the paucity of integrated data, the number of global annual deaths may exceed 500,000, with two-thirds of the mortality attributable to RHD and its complications^1,3,4^.

One of the major global health concerns regarding Strep A diseases is that the health and economic burden of Strep A infections are disproportionately concentrated in low- and middle-income countries. For example, acute rheumatic fever (ARF) rates remain high in resource-limited settings^1^. It has been reported that 42-60% of people with a history of ARF develop established RHD^5^. RHD, a cause of more than 300,000 deaths estimated in 2015 globally, is mostly concentrated in low-resource settings as three countries (India, Pakistan, and the Democratic Republic of the Congo) account for 73% of global cases^3^. Strep A pharyngitis is frequent among children, with an estimated rate of 22 episodes per 100 child-years^6,7^ and often observed in high income countries. Streptococcal pharyngitis, which is commonly self-limiting, can lead to various complications such as ARF^8^. Thus, superficial infections in the oropharyngeal mucosa and impetigo acting as the transmission reservoir should not be overlooked in acknowledging the importance of the prevention of Strep A infection^9^. Causal relationships between superficial and invasive Strep A diseases and post-streptococcal diseases have been identified as well^1,2^.

While no vaccine against Strep A exists yet, antibiotics such as oral and intramuscular penicillin have been widely used to treat patients with Strep A infections. For example, if a patient is confirmed positive for Streptococcal pharyngitis, antibiotics are prescribed to treat patients and prevent prognosis to ARF. In addition, intramuscular antibiotics can be used as a prophylaxis to prevent recurrent ARF. Nonetheless, existing prevention strategies may result in unnecessary consumption of antibiotics (i.e., false positives or prescription without any tests)^10^. A previous study also pointed out that it was challenging to increase patients’ compliance with the recommended schedule of injections over a long period of time^11^.

To address the unmet global health need for a vaccine against Strep A, the development of a Strep A vaccine has been discoursed and prioritized by the World Health Organization (WHO) in 2014, followed by the Product Development for Vaccines Advisory Committee (PDVAC) in 2016^12,13^. One of the aims of the Strep A Vaccine Global Consortium (SAVAC) is to evaluate the potential cost-effectiveness of a hypothetical Strep A vaccine to generate evidence useful for vaccine development and policy decision-making at the global- and national-levels. Given the absence of GAS vaccines and the scarcity of economic evaluations for GAS infections in broader geographical units, the main aim of the current study lies in estimating the cost-effectiveness of a hypothetical GAS vaccine against selected GAS infections and estimating the threshold cost per fully vaccinated person to be cost-effective by income group.

## Results

### Vaccine demand forecasts

**Fig. 1:**
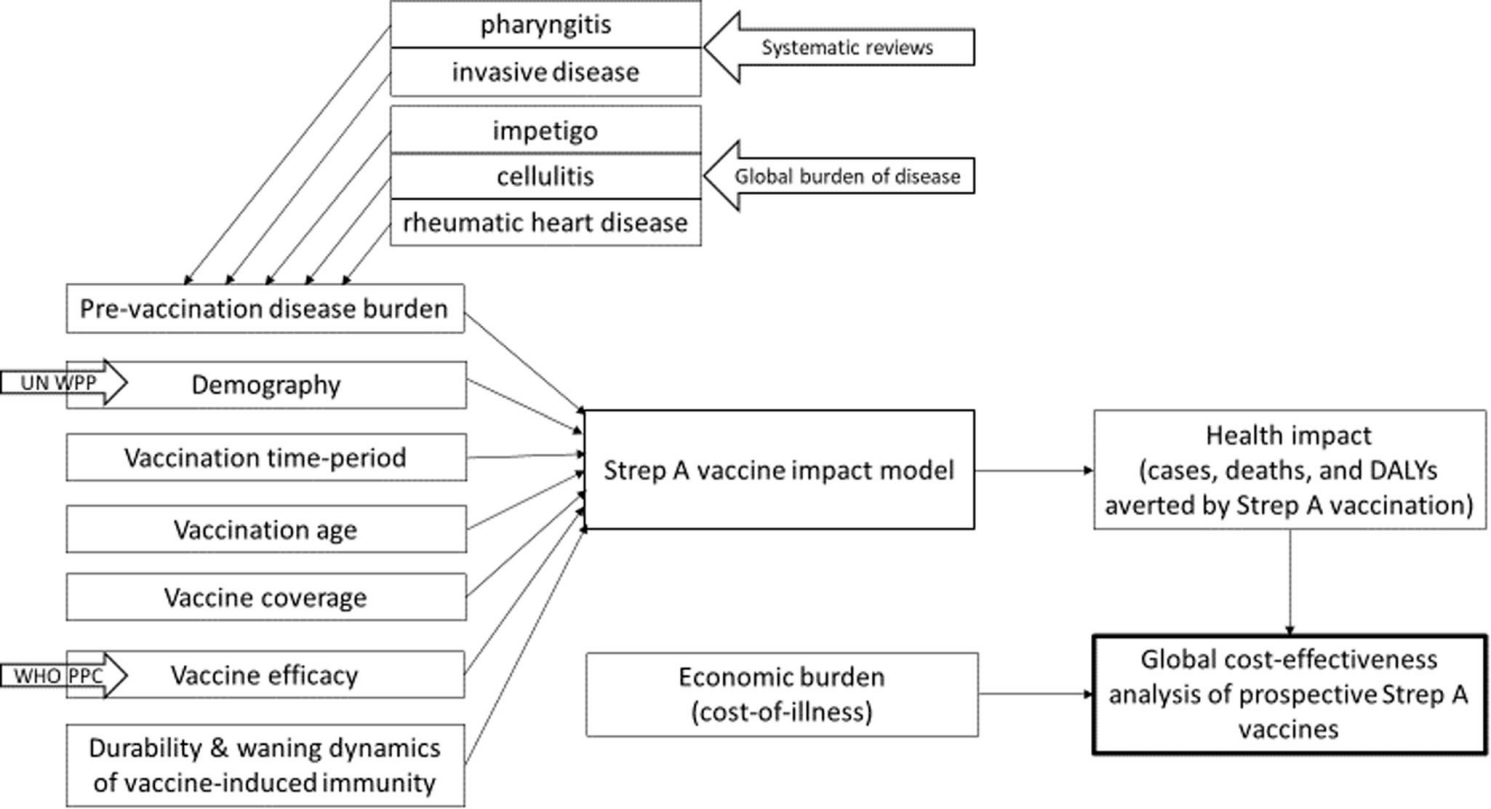
Cost-effectiveness modeling framework. The health impact estimates from the Strep A vaccine impact model and the cost-of-illness estimates from economic burden study are inputs to the global cost-effectiveness analysis of prospective Strep A vaccines. The figure is adapted from “Giannini F et al. Modeling the potential health impact of prospective Strep A vaccines. NPJ Vaccines, 2023^17^.

The cumulative number of vaccine doses required was calculated by income group and shown in Fig. 1. Overall, the total number of vaccine doses required for both routine vaccinations at age 0 year (at birth) and age 5 years was the greatest in LMICs followed by UMIC, LIC, and HIC. As expected, the cumulative vaccine demand was estimated to be the highest under scenarios 1 and 2 where country-specific coverage rates were assumed. There was no significant difference between routine vaccination at age 0 (at birth) and at age 5 years in terms of the number of vaccine doses required.

### Disability-adjusted life years averted

**Fig. 2:**
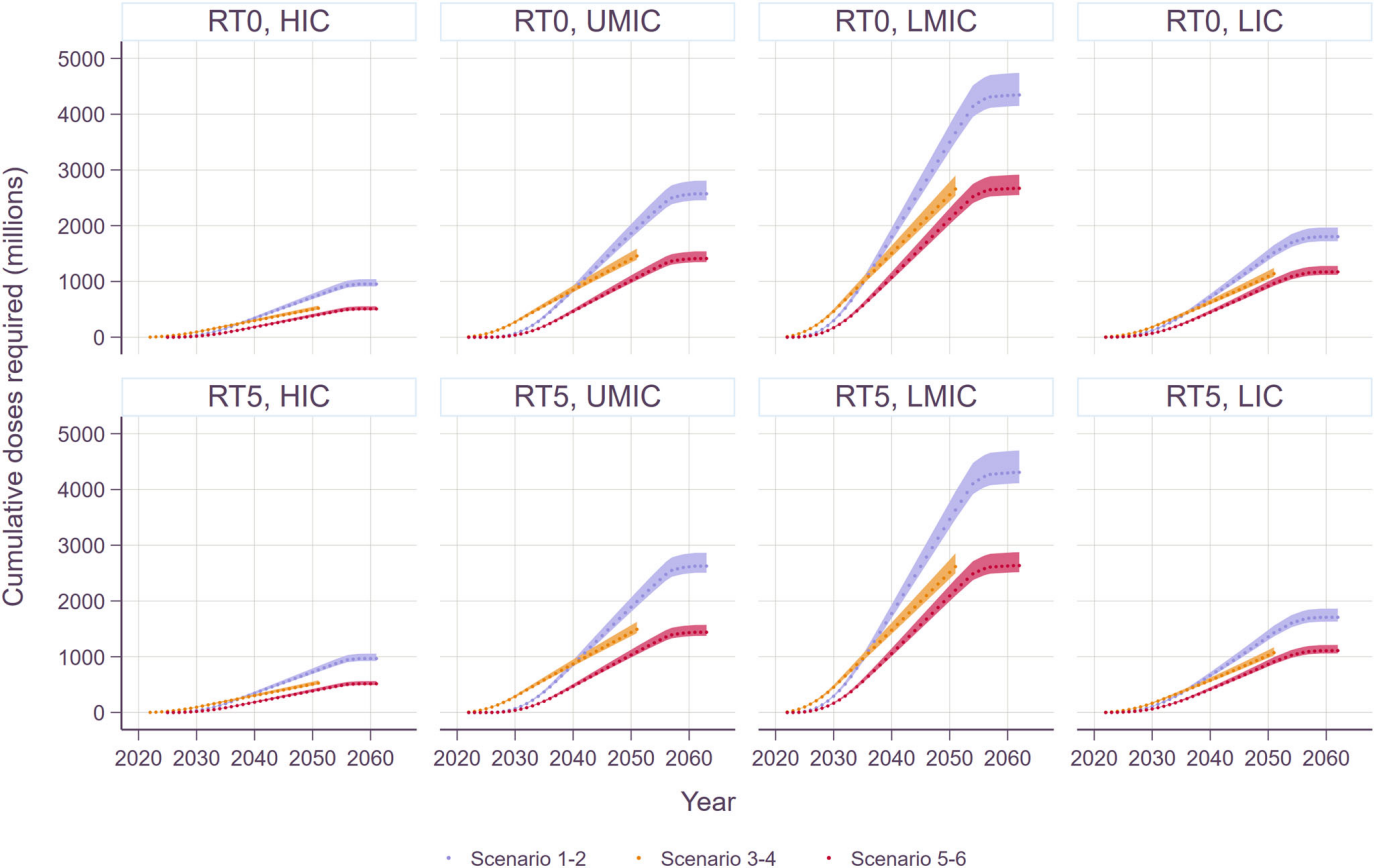
Cumulative number of Strep A vaccine doses required over time. RT0: routine vaccination for infants (at birth), RT5: routine vaccination at age 5 years. The upper and lower bounds are estimated based on 20% and 5% of wastage rates, respectively.

Disability-adjusted life years (DALYs) averted by vaccination are shown in Fig. 2. While the number of cases averted for pharyngitis and skin infections was far greater than for more severe illnesses such as RHD, or invasive infections, DALYs saved was higher for the severe illnesses than for superficial infections due to the longer duration of illness and higher disability weights, as well as premature deaths from more severe illnesses. As expected, vaccination scenarios with higher coverage rates such as scenarios 1 and 2 resulted in a greater number of averted DALYs than the rest of the scenarios where the peak coverage rates were identically assumed to be 50% across all countries. By income group, the highest number of DALYs averted was observed in LMICs regardless of the target age cohorts for routine vaccination. The number of DALYs averted for RHD was significantly lower in HIC than in other income groups. It should be noted that DALYs averted was sensitive to the choice of discounting rates (i.e., 0% vs. 3%), especially for chronic illnesses.

### Incremental cost-effectiveness ratios

**Fig. 3:**
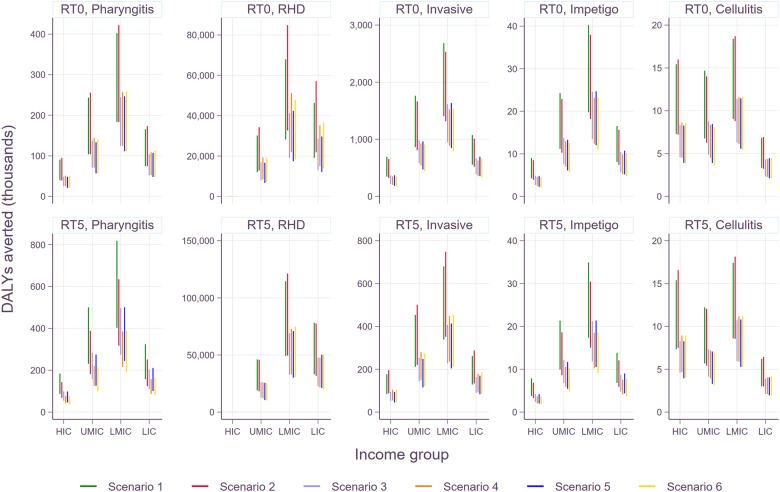
Averted DALYs by Strep A vaccination scenario and disease type by income group. RT0: routine vaccination for infants (at birth), RT5: routine vaccination at age 5 years. The upper bound of each bar shows health outcomes with no discounting (0%), whereas the lower bound estimates are based on the discount rate of 3%. Please note that scales on the Y-axes vary to improve readability across disease types.

The incremental cost-effectiveness ratios (ICERs) of the two routine vaccination strategies are shown by disease manifestation and income group in Fig. 3. While vaccinating the age cohort of 5 years old was more cost-effective than vaccinating infants for pharyngitis and RHD, this was the opposite for invasive infections. This is because the burden of pharyngitis was assumed to be more common in children 5–15 years old, and the incidence rate of RHD was also higher for children and adults (5–24 years old) than the cohorts younger than 5 years old. Thus, vaccinating the cohort of 5 years old averts a higher number of cases than vaccinating infants for pharyngitis and RHD. On the other hand, the incidence rate for invasive infections was estimated to be the highest among infants (0–12 months), making the infant routine vaccination more cost-effective than the 5-yo routine vaccination. In the case of impetigo and cellulitis, marginal differences were observed between the two routine vaccination strategies since the incidence rates of the two infections appeared to be quite consistent among the age cohorts affected by vaccination. Supplementary Table 1 shows all values to be cost-effective or cost-saving under different WTP thresholds.

### Threshold costs to be cost-effective

**Fig. 4:**
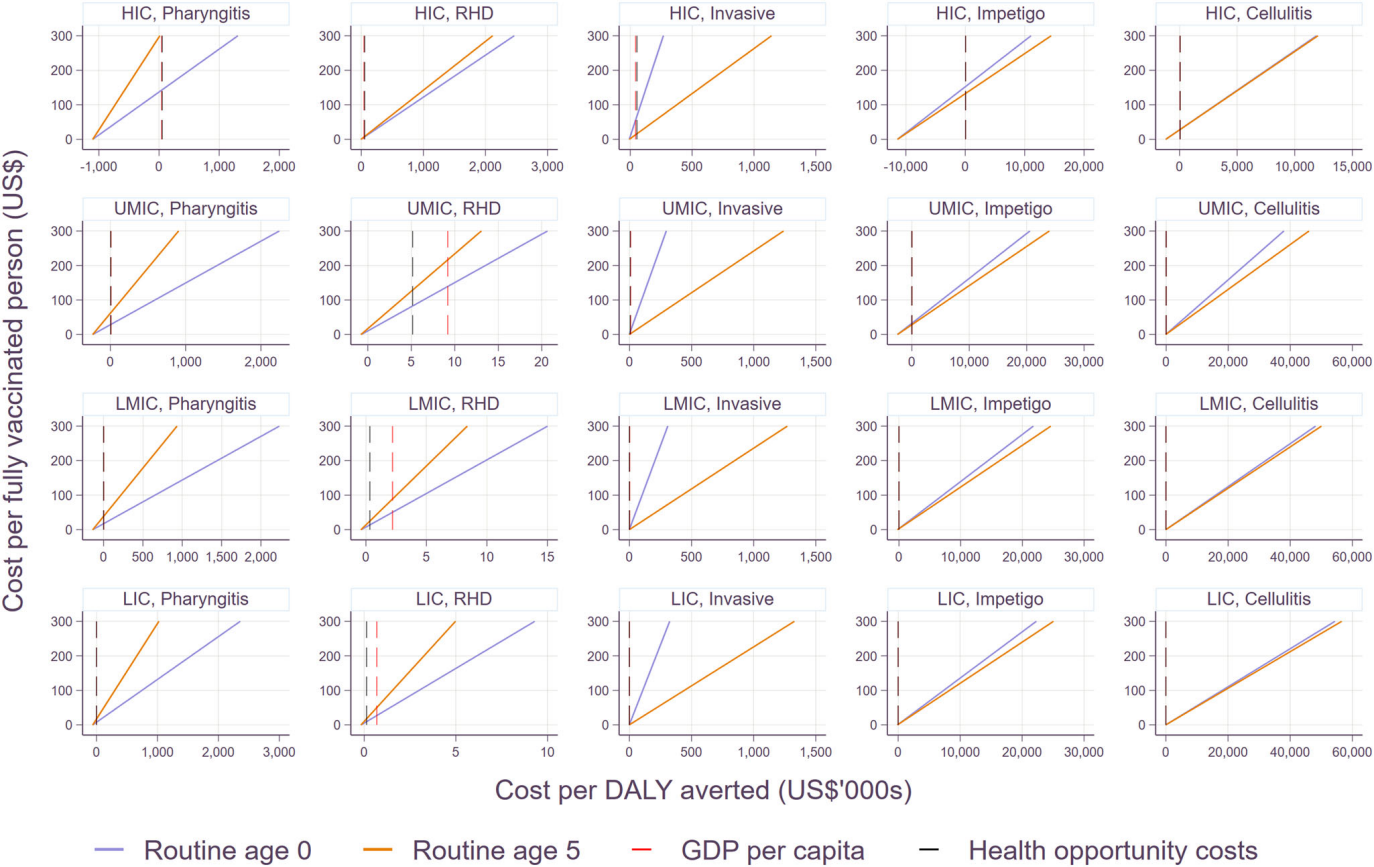
Incremental cost-effectiveness ratios by income group under scenario 1. Interventions are considered to be cost-effective if the total cost per fully vaccinated person is located on the left side of varying threshold costs (1xGDP per capita or health opportunity costs) per DALY averted.

Overall, vaccination would be cost-effective if the total cost per fully vaccinated person were set properly as shown in Fig. 4. In order for Strep A vaccination to be cost-effective at the threshold of 1× GDP per capita, the maximum vaccination cost per fully vaccinated person ranges from $8 to $308 for pharyngitis, $6 to $216 for RHD, $0.2 to $56 for invasive infections, $1 to $153 for impetigo, $0.1 to $28 for cellulitis, and $37 to $489 for all disease states combined. With the threshold of health opportunity costs, the total cost per fully vaccinated person was lower in non-HIC settings and higher in HIC: $7.6 to $311 for pharyngitis, $7 to $129 for RHD, $0.1 to $67 for invasive infections, $1.2 to $153 for impetigo, $0.1 to $29 for cellulitis, and $19 to $496 for all disease states combined. The threshold cost per fully vaccinated person were also presented with additional efficacy rate scenarios in Supplementary Figs. 2-4. As expected, the threshold cost per fully vaccinated person went down (less cost-effective) as efficacy rates decreased. In general, vaccination is more cost-effective in HIC for all disease types except RHD where the maximum cost per fully vaccination person is the highest in UMIC. It should be noted that the total vaccination cost per person needs to be set around $0.1 to $3 when only considering skin and invasive infections to be cost-effective in LMIC and LIC. However, the threshold cost per person is higher for pharyngitis and RHD, as well as for all disease states combined in LMIC and LIC.

**Fig. 5:**
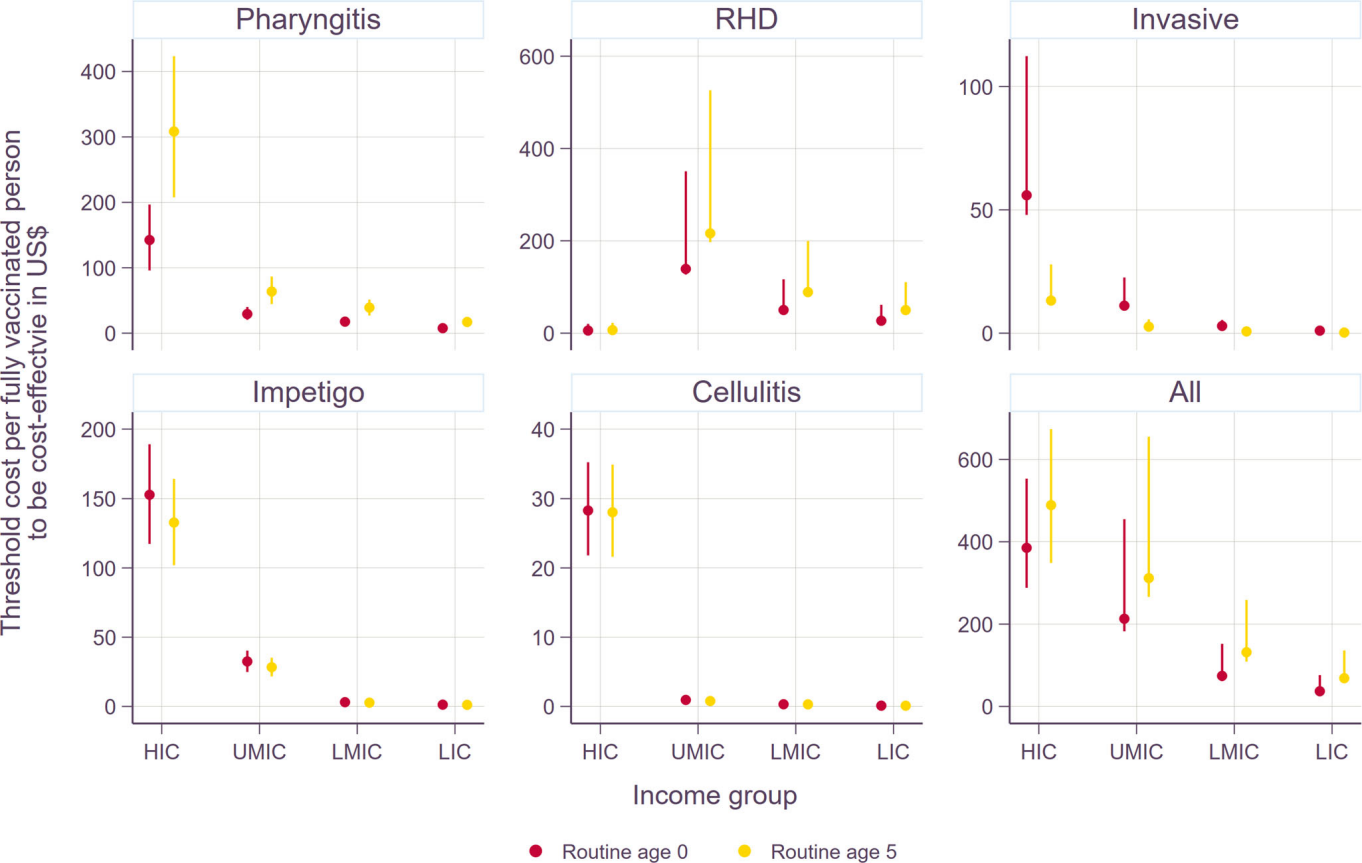
Threshold cost per fully vaccinated person to be cost-effective by income group under scenario 1. The estimates are based on 1× GDP per capita. The lower bounds are for the least favorable scenario: 20% wastage rate, lower bound of economic burden, and 3% discounting of health outcomes. The upper bounds are based on the most favorable scenario: 5% wastage rate, upper bound of economic burden, and 0% discounting of health outcomes. Please note that scales on the Y-axes vary to improve the readability across diseases. Please see Supplementary Fig. 1 for the rest of scenarios.

Figure 5 compares the threshold costs per fully vaccinated person among the six scenarios. Given the nature of a static model, the reduction in disease burden is linearly associated with vaccine efficacy, duration, and coverage rates. The waning dynamics of vaccine-derived immunity were modeled in two ways: (i) vaccine-induced immune protection at maximum efficacy for 10 years and null thereafter and (ii) waning linearly with an annual reduction in efficacy equivalent to 5% of maximum efficacy for 20 years and null thereafter (i.e., waning to 50% of maximum efficacy after 10 years). Since the overall vaccine protection for these two waning dynamics of vaccine-derived immunity is similar, the ICER estimates are similar among the six scenarios. If any, marginal differences could be observed mainly due to the background demographic information (i.e., varying mortality rates by year).

**Fig. 6:**
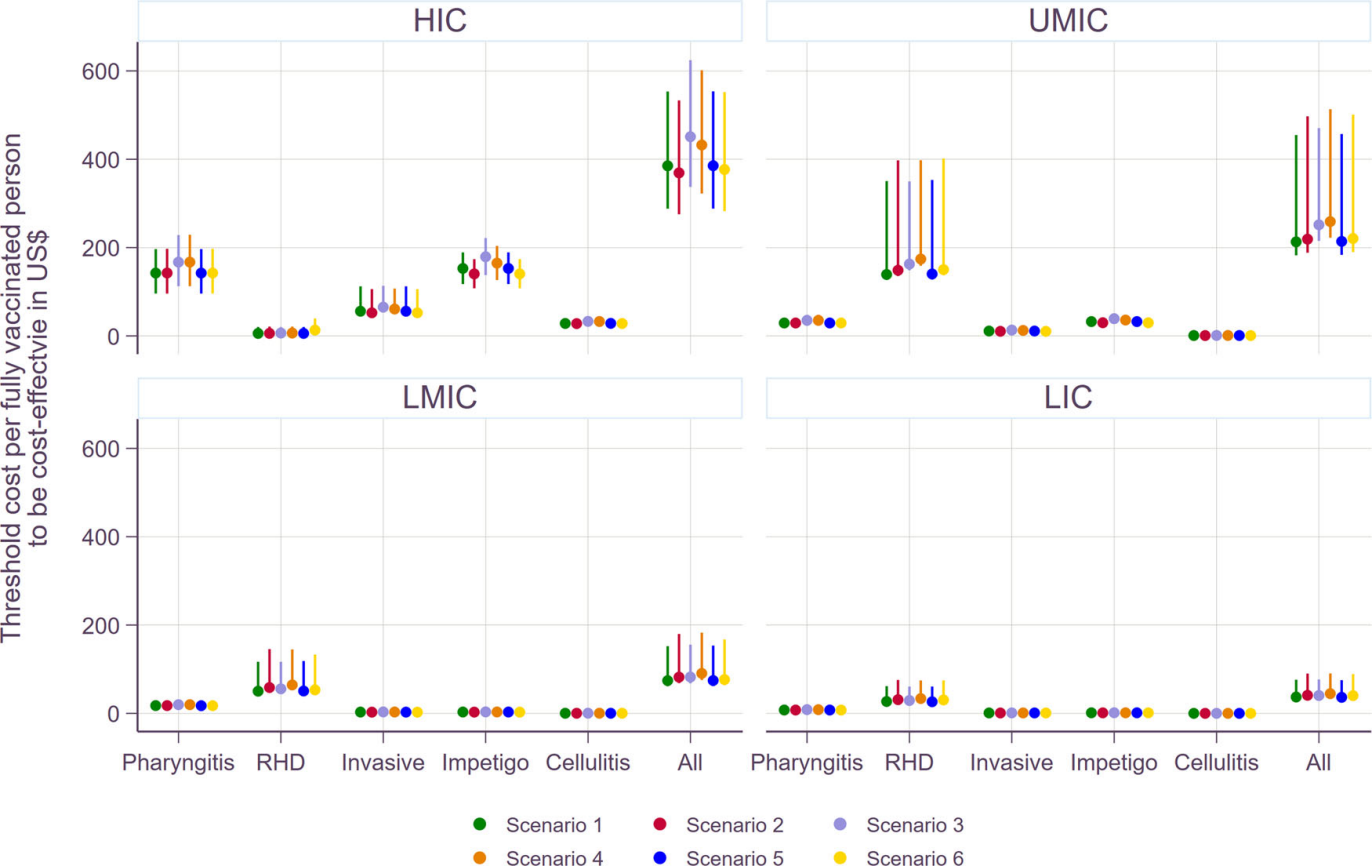
Threshold cost per fully vaccinated person to be cost-effective by scenario. The lower bounds are based on the least favorable scenario: 20% wastage rate, lower bound of economic burden, and 3% discounting of health outcomes. The upper bounds are for the most favorable scenario: 5% wastage rate, upper bound of economic burden, and 0% discounting of health outcomes.

**Fig. 7:**
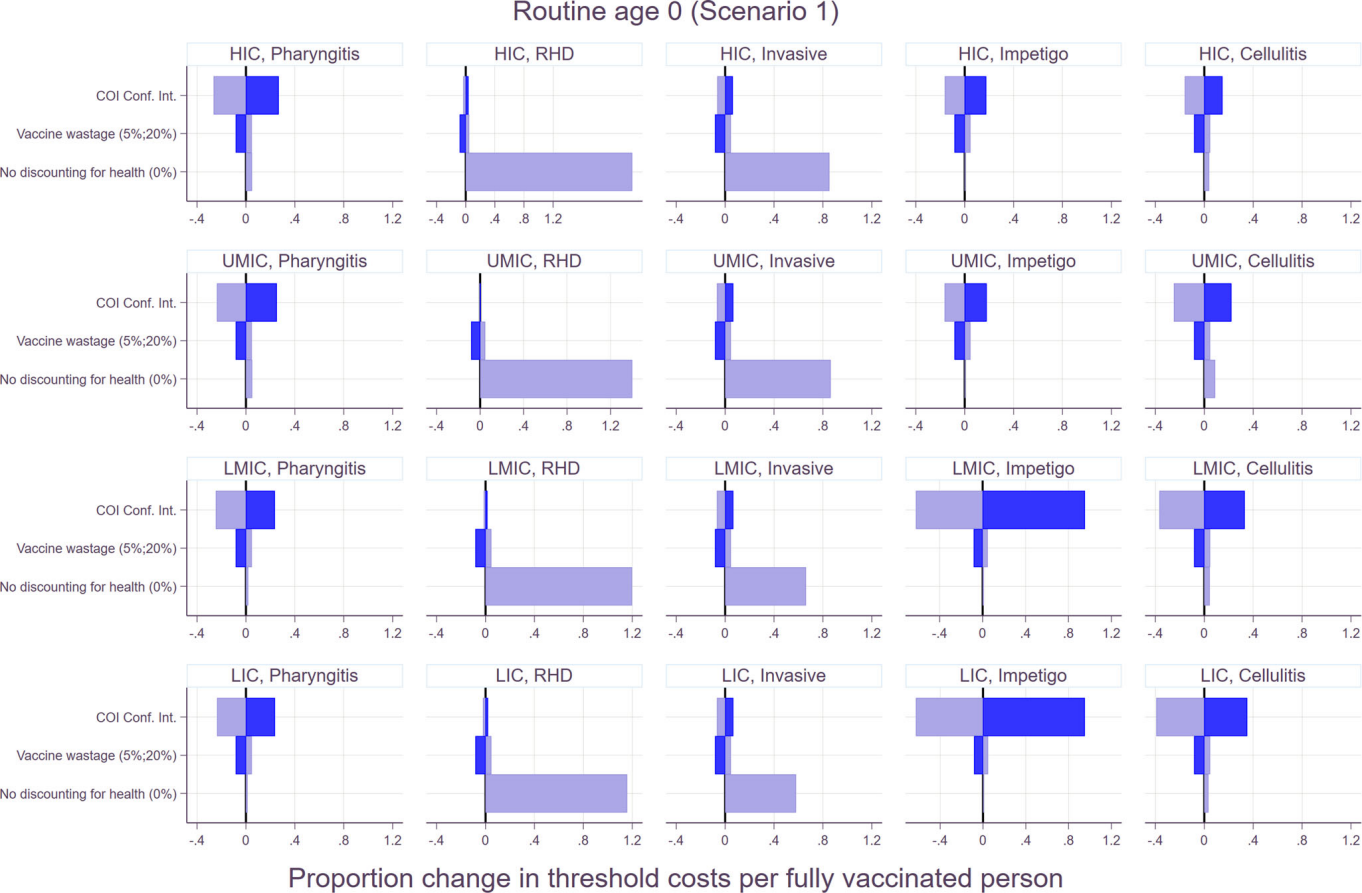
Univariate sensitivity analysis for routine vaccination at age 0 (at birth) under scenario 1. The similar patterns were observed for routine vaccination at age 5 years and other scenarios as well. Please see Supplementary Fig. 5 for the rest of the scenarios.

Both univariate and multivariate sensitivity analyses were carried out and shown in Figs. 6 and 7. In the univariate sensitivity analysis, the changes in the economic burden had the most significant impact on the proportion change in threshold costs per fully vaccinated person for superficial diseases including pharyngitis and skin infections. On the other hand, the change in the threshold costs was more sensitive to the choice of discounting for health outcomes for chronic and severe illnesses such as RHD, and invasive infections. In the multivariate analysis, the impact of no discounting for health outcomes outweighed the changes in the other indicators for RHD and invasive infections. In other words, regardless of the variations in the economic burden and wastage rates, the threshold costs per fully vaccinated person increased if there was no discounting for health outcomes for RHD and invasive infections. However, applying the lower bound of the economic burden resulted in the decrease in the total vaccination cost per person for pharyngitis and skin infections even if health outcomes were not discounted.

## Discussion

The present study investigated the cost-effectiveness of a hypothetical Strep A vaccine at the global-level. A previous review study reported that existing CEA studies on Strep A were limited in number and disproportionately lower in the LMIC and LIC settings compared to the HIC and UMIC settings^10^. In particular, despite a wide range of disease manifestations caused by Strep A, the types of clinical outcomes considered in the majority of existing CEA studies were limited. In the current analysis, it was found that Strep A vaccination would be cost-effective if the maximum cost per fully vaccinated person were properly set according to the income group considered: $8 to $308 for pharyngitis, $6 to $216 for RHD, $0.2 to $56 for invasive infections, $1 to $153 for impetigo, $0.1 to $28 for cellulitis, and $37 to $489 for all disease states combined, at the threshold of 1× GDP per capita. The values were lower (more conservative) in non-HIC settings if health opportunity costs were used as thresholds. In HIC, vaccination would be particularly cost-effective for superficial infections including pharyngitis and skin infections, as well as for invasive infections. In LMIC and LIC, the total vaccination cost per person would need to be set lower than that in HIC and UMIC in order for vaccination to be cost-effective. However, vaccination would be more cost-effective to prevent RHD in LMIC and LIC than in HIC due to the high burden of RHD estimated in LMIC and LIC. Overall, vaccination would be an effective prevention strategy considering the impact of vaccination on all five disease outcomes combined. It should be also noted that an additional health benefit of GAS vaccines would be reduction in global antimicrobial resistance (AMR) risk. It is known that Strep A is a pathogen listed by the US CDC as a priority for AMR^14^ and Strep A vaccination could lower antibiotic consumption by lowering the incidence of Strep A pharyngitis. Lewnard et al. estimated that an 80%-effective Strep A vaccine could prevent between 5.4% and 17.1% of all outpatient antibiotic prescriptions for children aged 3–9 years in the United States^15^. Given that both vaccine procurement and delivery costs were not available due to the absence of GAS vaccines, the maximum total cost per fully vaccinated person to be cost-effective was estimated. Thus, the maximum cost for administering one dose can be approximately calculated by dividing the maximum total cost per fully vaccinated person by 3.

Some areas of uncertainty deserve attention. Given that there is no Strep A vaccine available yet, the current study followed the WHO preferred product characteristics for a hypothetical Strep A vaccine. While this could be a reasonable approach in the absence of actual Strep A vaccines, the study outcomes are sensitive to vaccine characteristics such as efficacy, waning, and duration of protection. These factors will need to be updated as clinical trials for potential vaccine candidates advance. Similarly, given the absence of any large clinical trials or post-introduction studies to detect safety signals, the impact of adverse events following vaccination was excluded. Adverse events following immunization typically cause additional healthcare costs, which in turn, makes the introduction of vaccination less cost-effective. Nonetheless, the recent article by Asturias et al. highlighted that no clinical or biological safety signals were detected in any of the five early phase trials in the modern era^16^. None of the participants in the trials developed clinical, echocardiographic or laboratory evidence of rheumatogenicity or nephritogenicity. It should be noted that the vaccine impact model did not yet consider the etiological pathway between infection and ARF and RHD. Thus, it would be likely that some proportion of incident RHD among children > 5 years old would be prevented by an infant vaccination scenario, since prior infections would be prevented. Prior to conducting the current CEA analysis, the disease and economic burden of Strep A infections were estimated at the global-level and presented separately^17,18^. While both studies were carried out based on extensive literature reviews, meta-analyses, and extrapolations, it should be noted that the number of primary data sources was scarce particularly in LMIC and LIC. Considering that the burden of Strep A infections and the costs of illnesses are critical inputs for a cost-effectiveness analysis, future research is needed to increase a number of primary data points such as surveillance activities, and field-based economic burden studies, paying special attention to the lack of evidence in LMIC and LIC. Lastly, every country will have different health system capacity and costs associated with deploying a vaccine. Given that both vaccine procurement and delivery cost were unknown, the threshold cost (procurement and implementation) per fully vaccinated person to be cost-effective was estimated, instead of setting up additional assumptions on the procurement costs and variable costs of implementing a new vaccine.

Strep A can be transmitted from host to host during an episode of acute disease. The previous review study reported that none of the existing CEA studies included the indirect benefits from reducing Strep A transmission^10^. In addition, oral or intramuscular penicillin has been widely used to reduce the disease progression and treat the symptoms. While the use of such antibiotics has proved effective, it is known that there is a growing concern about excessive antibiotic uses resulting in antimicrobial resistance^15,19–21^. It may be premature to include all these factors into CEA frameworks at once given the lack of available evidence. Nonetheless, they should be included in the domain of Strep A discussions since the reductions in host-to-host transmission and antimicrobial resistance are additional benefits that the development of safe and efficacious vaccines will bring to the society.

## Methods

### Model structure and assumptions

**Fig. 8:**
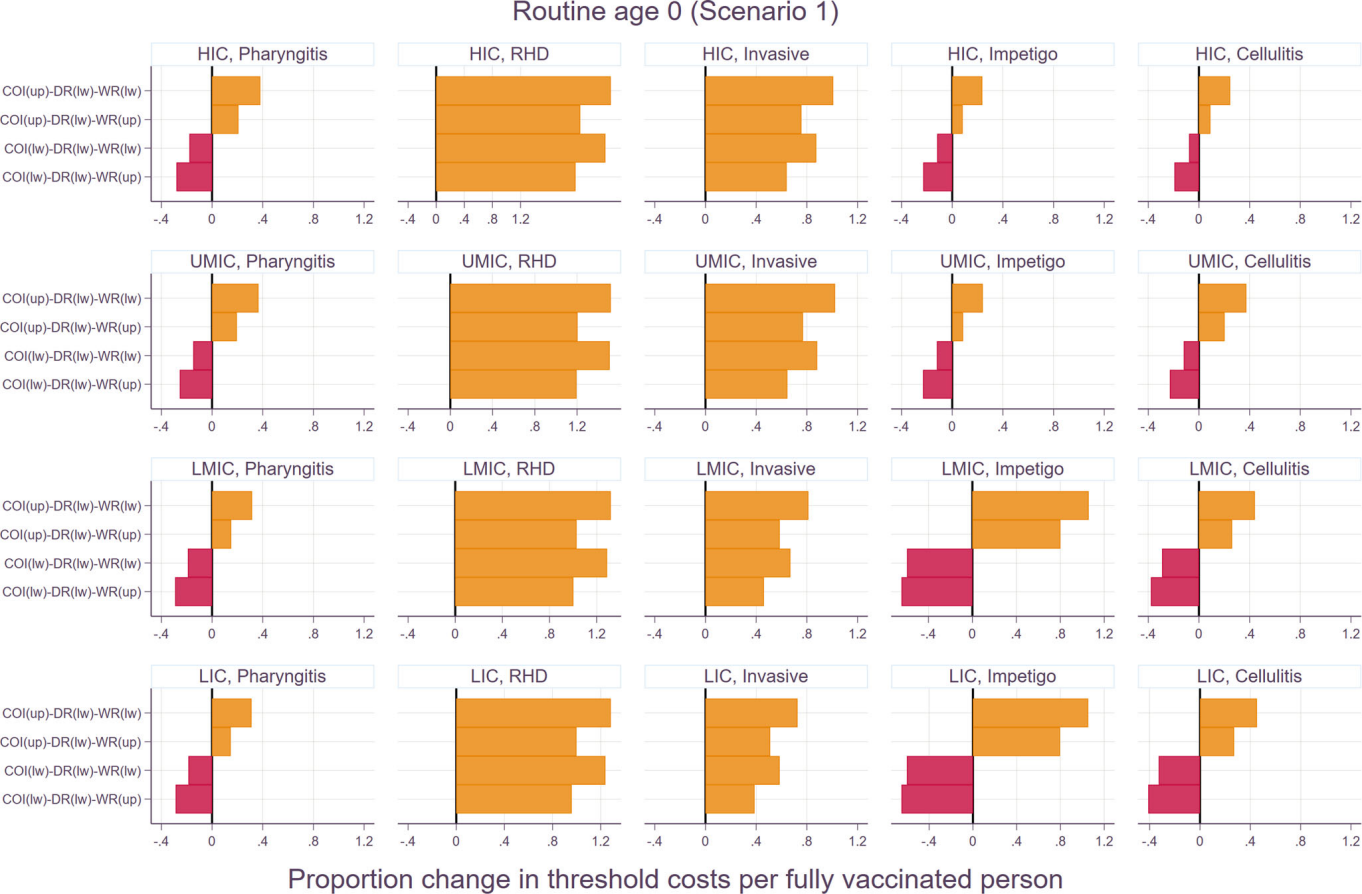
Multivariate sensitivity analysis for routine vaccination at age 0 (at birth) under scenario 1. COI (up): the upper bound of the confidence interval, COI (lw): the lower bound of the confidence interval, DR (lw): no discounting for health outcomes, WR (up): 20% wastage rate, WR (lw): 5% wastage rate. The similar patterns were observed for routine vaccination at age 5 years and other scenarios as well. Please see Supplementary Fig. 6 for the rest of scenarios.

The current study investigates the cost-effectiveness of a hypothetical Strep A vaccine at the global-level and presents outcomes by income group as classified by the World Bank: high-income (HIC), upper-middle-income (UMIC), lower-middle-income (LMIC), and low-income countries (LIC). Among a wide range of disease manifestations caused by Strep A, five clinical outcomes were selected for the current cost-effectiveness analysis: pharyngitis, RHD, invasive infections, impetigo, and cellulitis. The details on the economic burden estimation and the impact of vaccination on disease burden are described elsewhere^17,18^. The overall structure of the model is shown in Fig. 8. Briefly, the economic burden of the five disease types was estimated including direct medical cost (DMC), direct non-medical cost (DNMC), indirect cost (IC), and productivity loss due to premature death from RHD or invasive infections. Each of these cost components was estimated at the country-level using adjustment factors based on observed values (existing literature), the WHO-CHOICE database, GDP per capita, and minimum wage, acknowledging the insufficient number of the existing economic burden studies for multiple disease manifestations caused by GAS across the four income groups. A probabilistic multivariate sensitivity analysis was extensively carried out for the economic burden of GAS infections. The minimum and maximum values of the DMC and DNMC adjustment factors, as well as of the duration of illness for each disease category were utilized to construct the distributions by income group. In addition, the lower- and upper-bounds of the weighted average age of death were estimated and utilized to address uncertainty surrounding productivity loss due to premature death from RHD and invasive infections. A Monte Carlo simulation was carried out based on 5,000 random draws for input parameters to estimate 95% confidence intervals. The upper- and lower-bounds of the point estimates which vary by disease outcome and by income group were used for the univariate and multivariate sensitivity analyses in the current CEA study. The pre-vaccination disease burden was estimated at the country-level based on country- and age-specific incidence rates for cellulitis and RHD and global age-specific prevalence for impetigo from the 2019 Global Burden of Disease (GBD) study. Systematic reviews were carried out as part of the SAVAC project to estimate the pre-vaccination disease burden for invasive diseases and pharyngitis^7^. Vaccination impact was estimated based on a static cohort model, and six vaccination scenarios were considered by mixing a combination of the following components as shown in Table 1: (1) vaccine adoption years (varying adoption years by country or the same adoption year (2022) for all countries), coverage rates (country-specific *Haemophilus influenza* type B vaccine (Hib3) coverage rates or the coverage rate of 50% for all countries), the duration of vaccine-derived immunity (full efficacy for 10 years or linear waning over 20 years). The WHO preferred product characteristics (PPC) for a Strep A vaccine were followed to set up assumptions on target age cohorts for routine vaccination and vaccine efficacy rates which vary by disease type^9,22^: pharyngitis (80%), RHD (50%), invasive infections (70%), impetigo (80%), and cellulitis (70%). The initial vaccination coverage rate was assumed to be 10% of the peak coverage rates, and the annual uptake rate of 10% was applied since the year of vaccine introduction. Vaccination impact was estimated for the lifetime health benefits of vaccination at birth or 5 years of age for 30 cohorts from the year of vaccine introduction^17^. Considering that there is no supporting biological or clinical justification for a hypothetical GAS vaccine yet, we developed the Strep A vaccine impact model using the R statistical software and included a user-friendly R Shiny web application. The program code and data for the vaccine impact model is available as an R package, GAS Impact Model (https://github.com/fionagi/GASImpactModel), and modeling analysis can be conducted through the R Shiny web application (https://github.com/fionagi/GASImpactModel_App). Through the web application, the impact of a selected vaccination scenario can be visualized for any of 205 countries. The app shows the predicted lifetime health benefits from age of vaccination associated with the vaccination of multiple cohorts. Key input values including the duration of protection can be easily adjusted in this application. The vaccine impact model can estimate the health benefits of vaccination by calendar year, birth year, and year of vaccination.

**Table 1:** Health economic parameters.

Item	Assumption
Geographical presentation	World Bank income groups (HIC, UMIC, LMIC, LIC)
Vaccine doses	3 doses
Vaccination strategies	Routine vaccination at birth or 5 years of age
Cost per fully vaccinated person	$0–$300
Discounting	3% discounting for costs and health outcomes (default); 3% discounting for costs and 0% discounting for health outcomes (sensitivity analysis)
Wastage factor during vaccination campaigns	10% (default); 5% and 20% (sensitivity analysis)
Economic burden	Point estimates (default); 95% Confidence Intervals (sensitivity analysis); societal perspective
Cost-effectiveness threshold	1 x GDP per capita (default); health opportunity costs (health care system threshold)
Time period	Lifetime impact among 30 cohorts from the year of vaccine introduction
Vaccination scenarios	Scenario 1 - vaccine adoption year (country-specific), coverage rate (country-specific Hib3), full efficacy for 10 years
	Scenario 2 - vaccine adoption year (country-specific), coverage rate (country-specific Hib3), linear waning over 20 years
	Scenario 3 - vaccine adoption year (2022), coverage rate (50%), full efficacy for 10 years
	Scenario 4 - vaccine adoption year (2022), coverage rate (50%), linear waning over 20 years
	Scenario 5 - vaccine adoption year (country-specific), coverage rate (50%), full efficacy for 10 years
	Scenario 6 - vaccine adoption year (country-specific), coverage rate (50%), linear waning over 20 years

As shown in Table 1, future costs and health outcomes were discounted at the rate of 3%, but health outcomes with no discounting were also considered following the WHO guideline^23^. The number of vaccine doses required was estimated over a 30-year period by vaccination scenario and varying wastage rate assumptions. Both vaccine procurement and delivery costs are unknown since there is no vaccine available yet against Strep A infections. Instead of setting up additional assumptions on vaccination costs, a range ($0 - $300) of the total cost per fully vaccinated person is applied^24,25^, and the maximum cost per fully vaccinated person to be cost-effective is derived at varying threshold costs per DALY averted. Given that the conventional threshold approach (i.e., 3 times GDP per capita) has been criticized and subsequently discouraged by the WHO^26,27^, population weighted cost per DALY averted which takes into account marginal productivity of healthcare expenditure (health opportunity cost) is considered in addition to the conventional willingness-to-pay threshold per DALY averted (1 x GDP per capita)^28^.

### Sensitivity analysis

Considering a degree of uncertainty in the current analysis, deterministic sensitivity analysis was carried out. Both univariate and multivariate sensitivity analyses were conducted. In the univariate sensitivity analysis, the following indicators were investigated: the economic burden for each of the disease manifestations, wastage rates during vaccination campaign, and discounting for health outcomes. For the economic burden, the 95% confidence intervals were estimated based on a Monte Carlo simulation, and the lower and upper bounds of the intervals were used for the sensitivity analysis. Wastage rate creates another source of uncertainty as wastage-level would be variable by country during vaccination campaigns. In addition to the default value of 10%, 5%, and 20% of wastage rates were considered. Discounting health outcomes have been widely discussed, and the current analysis included no discounting for health outcomes in addition to the default rate of 3%. For the multivariate sensitivity analysis, all three indicators were varied simultaneously. Lastly, given that the vaccine efficacy rates taken from the WHO PPC were hypothetical, additional efficacy scenarios were considered: 20%, 40%, and 60% reduction of the default efficacy rates which were variable by disease manifestation.

### Reporting summary

Further information on research design is available in the Nature Research Reporting Summary linked to this article.

### Supplementary information

Supplementary Information

Reporting Summary

## Data Availability

The datasets analyzed in the current study are open to public users.

## Code availability

https://github.com/fionagi/GASImpactModel.
