# Supplementary material for: The potential global cost-effectiveness of prospective Strep A vaccines and associated implementation efforts
Source: NPJ Vaccines. 2023 Aug 25;8:128. doi: 10.1038/s41541-023-00718-7 (PMC10457324; doi:10.1038/s41541-023-00718-7)
Supplement: Supplementary file 1 — Supplementary Information [file 41541_2023_718_MOESM1_ESM.pdf]

Supplementary information

Supplementary Table 1. Threshold costs per fully vaccinated person to be cost-effective and cost-saving (default efficacy rates scenario)

Income group	RT Age cohort	Threshold cost per fully vaccinated person																	
		To be cost-effective at varying thresholds												To be cost-saving					
		Health opportunity costs						GPD per capita											
		Pharyngitis	RHD	Invasive	Impetigo	Cellulitis	All	Pharyngitis	RHD	Invasive	Impetigo	Cellulitis	All	Pharyngitis	RHD	Invasive	Impetigo	Cellulitis	All
HIC	rt0	$143.7	$7.0	$67.1	$152.9	$28.5	$399.3	$142.4	$5.8	$55.9	$152.8	$28.3	$385.2	$137.0	$0.4	$8.0	$152.2	$27.3	$324.9
HIC	rt5	$311.0	$8.2	$15.9	$132.9	$28.3	$496.3	$308.3	$6.7	$13.2	$132.8	$28.0	$489.0	$296.4	$0.5	$1.7	$132.3	$27.0	$458.0
UMIC	rt0	$28.8	$82.6	$7.1	$32.5	$0.9	$151.8	$29.3	$139.2	$11.2	$32.5	$0.9	$213.1	$28.2	$10.3	$1.9	$32.4	$0.9	$73.6
UMIC	rt5	$62.6	$128.7	$1.7	$28.3	$0.8	$222.0	$63.7	$216.3	$2.6	$28.3	$0.8	$311.7	$61.3	$16.8	$0.4	$28.2	$0.7	$107.5
LMIC	rt0	$17.4	$13.8	$1.1	$3.1	$0.3	$35.7	$17.7	$50.3	$2.9	$3.1	$0.3	$74.3	$17.4	$7.4	$0.8	$3.1	$0.3	$29.0
LMIC	rt5	$38.6	$25.0	$0.3	$2.7	$0.3	$66.9	$39.1	$88.9	$0.7	$2.7	$0.3	$131.8	$38.5	$13.8	$0.2	$2.7	$0.3	$55.4
LIC	rt0	$7.6	$9.3	$0.5	$1.3	$0.1	$18.8	$7.7	$26.9	$1.0	$1.3	$0.1	$37.1	$7.6	$5.1	$0.4	$1.3	$0.1	$14.5
LIC	rt5	$17.0	$17.6	$0.1	$1.2	$0.1	$36.0	$17.1	$50.0	$0.2	$1.2	$0.1	$68.7	$16.9	$9.9	$0.1	$1.2	$0.1	$28.2

Supplementary Figure 1. Threshold cost per fully vaccinated person to be cost-effective by income group

Scenario 2

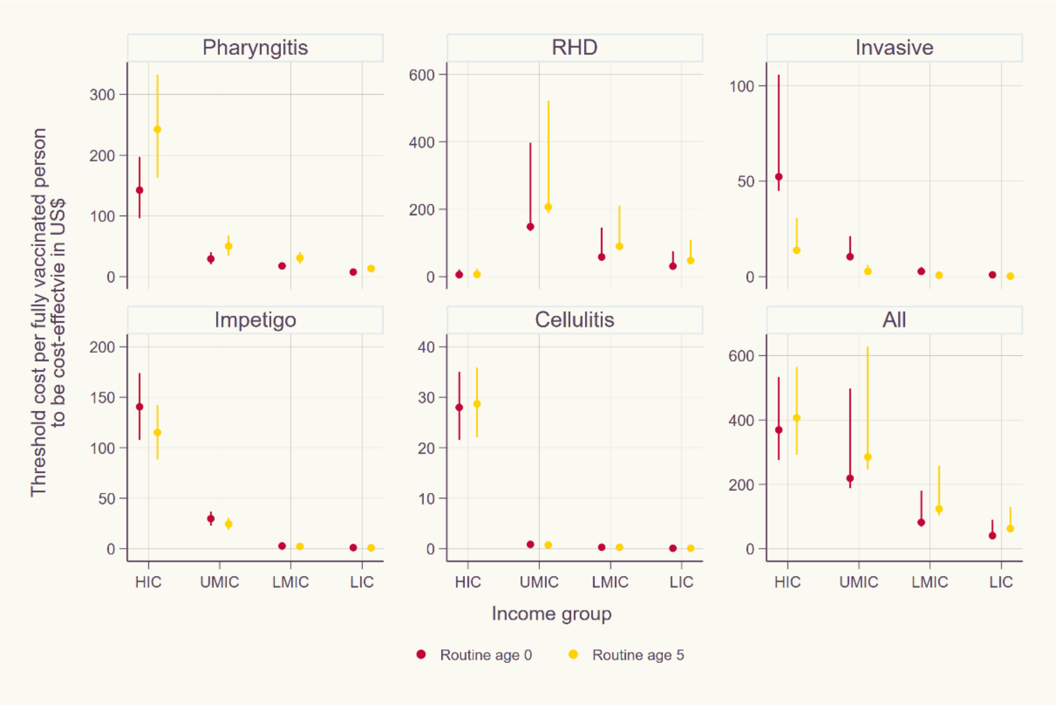

Scenario 3

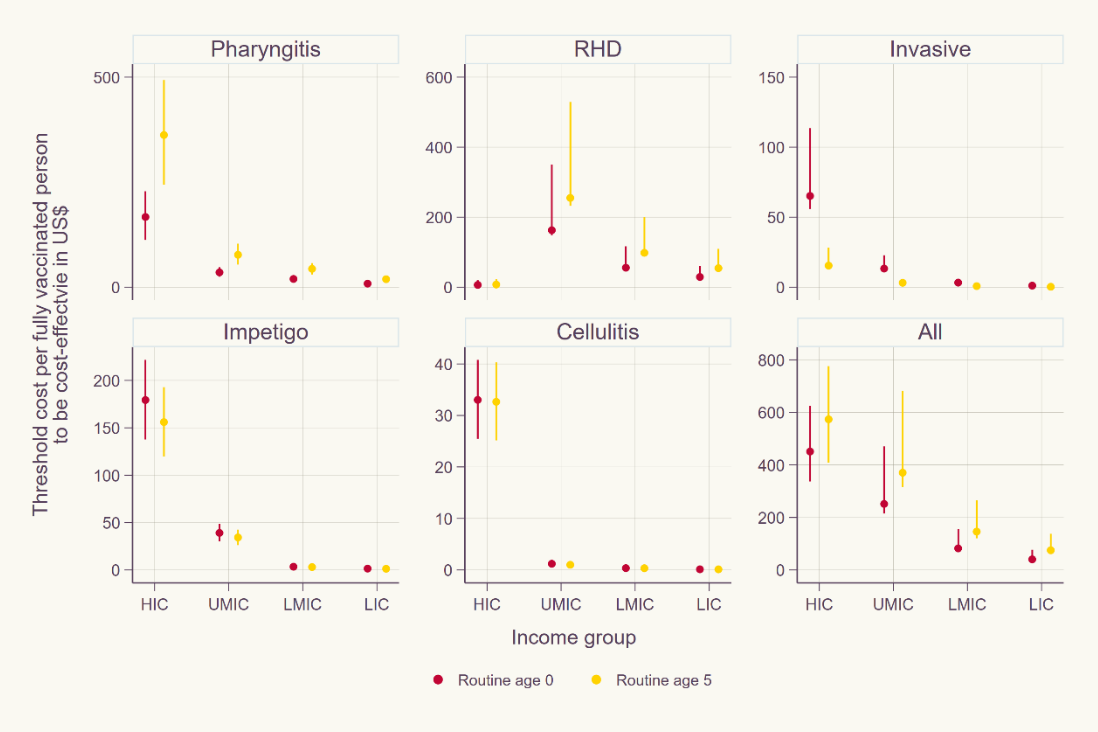

Scenario 4

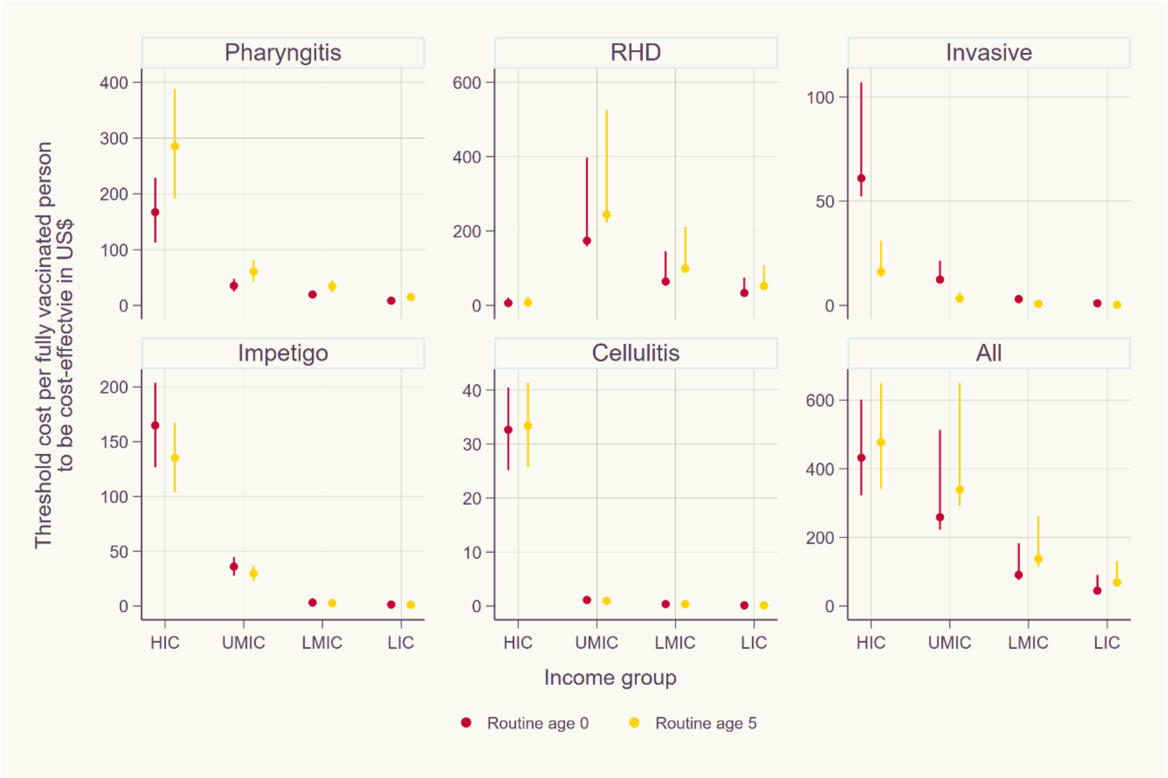

Scenario 5

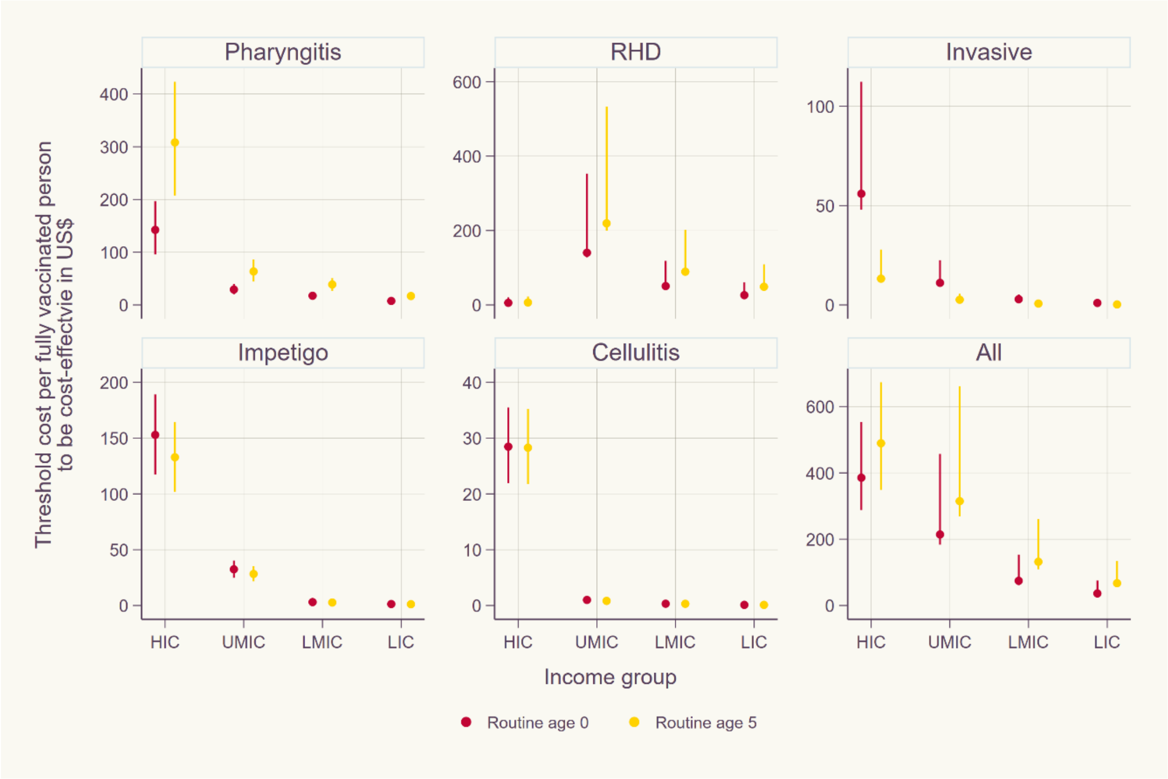

Scenario 6

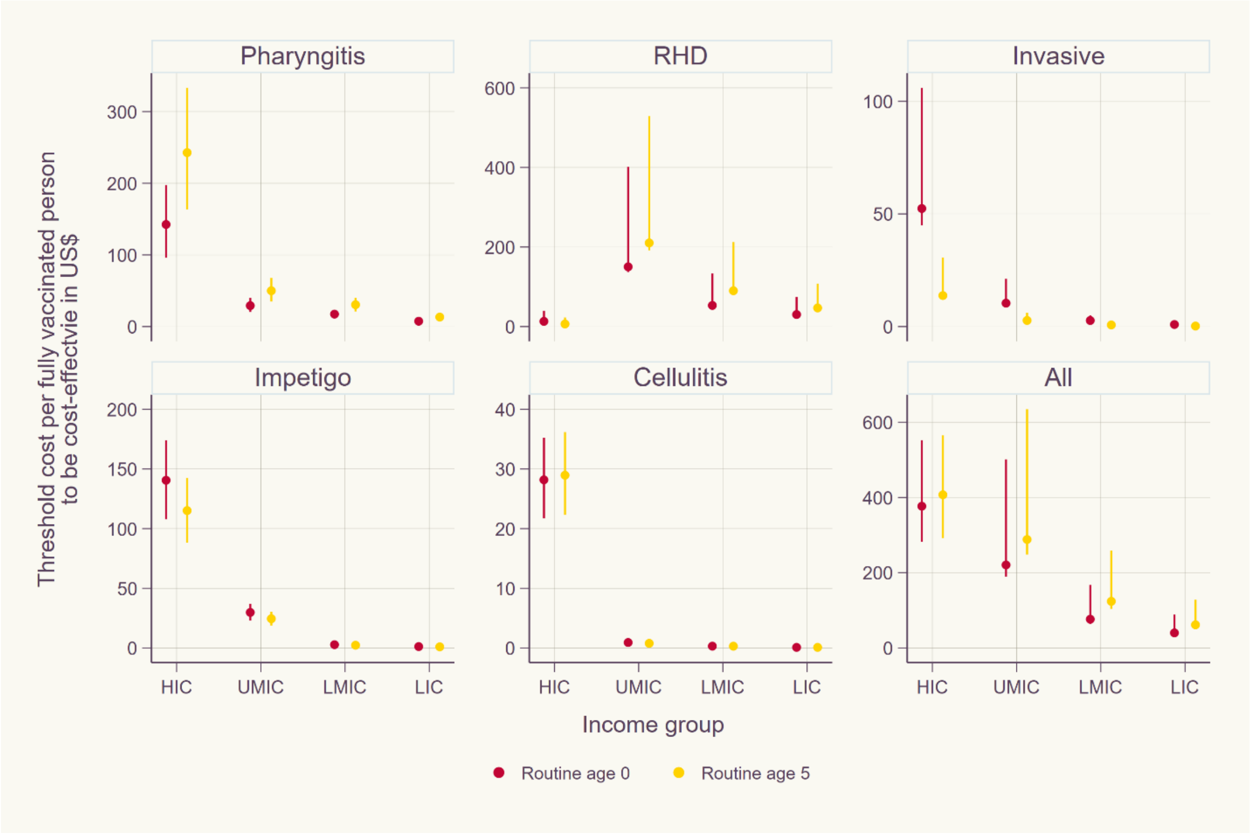

Pharyngitis
RHD
Invasive
Impetigo
Cellulitis
All
Threshold cost per fully vaccinated person to be cost-effectvie in US$
HIC
UMIC
LMIC
LIC
Income group
Routine age 0
Routine age 5

Supplementary Figure 2. Threshold cost per fully vaccinated person to be cost-effective by scenario: 20% reduction of the default efficacy rate scenario

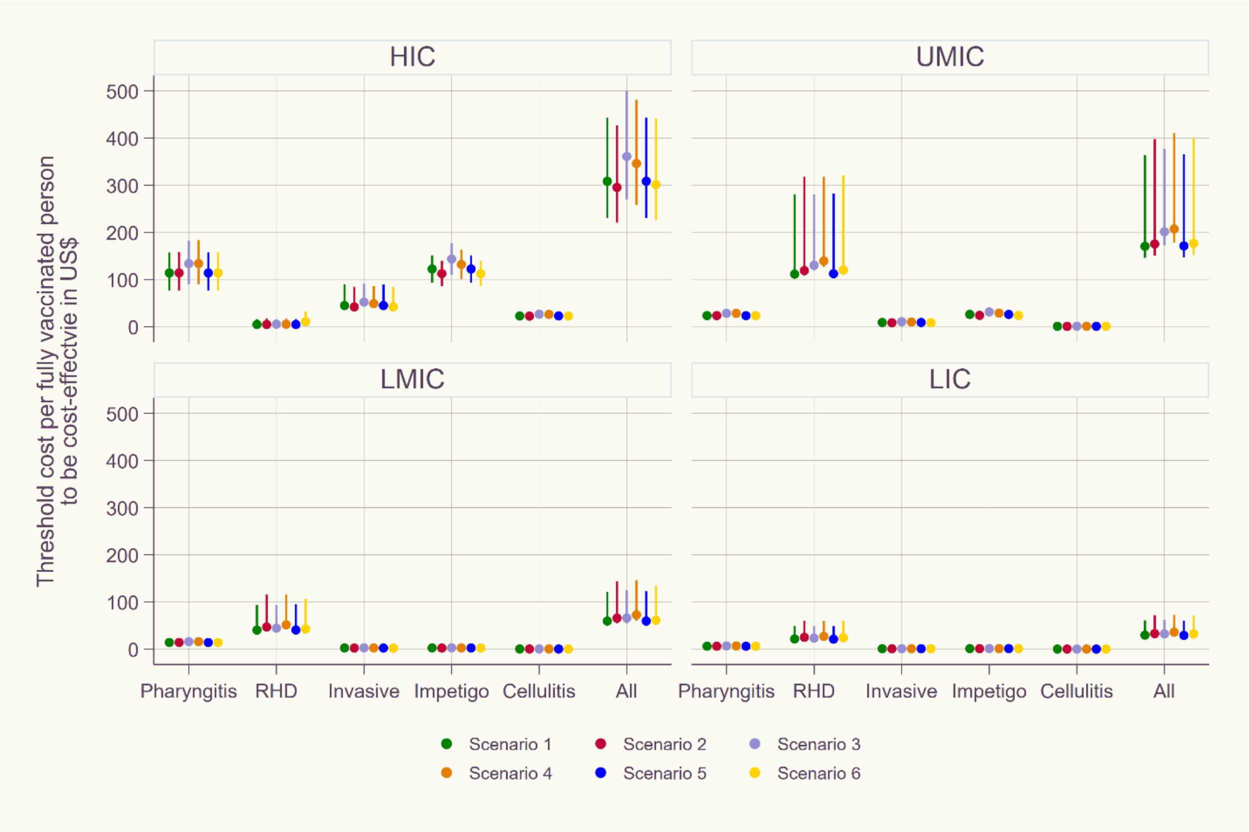

The lower bounds are based on the least favorable scenario: 20% wastage rate, lower bound of economic burden, and 3% discounting of health outcomes. The upper bounds are for the most favorable scenario: 5% wastage rate, upper bound of economic burden, and 0% discounting of health outcomes.

Supplementary Figure 3. Threshold cost per fully vaccinated person to be cost-effective by scenario: 40% reduction of the default efficacy rate scenario

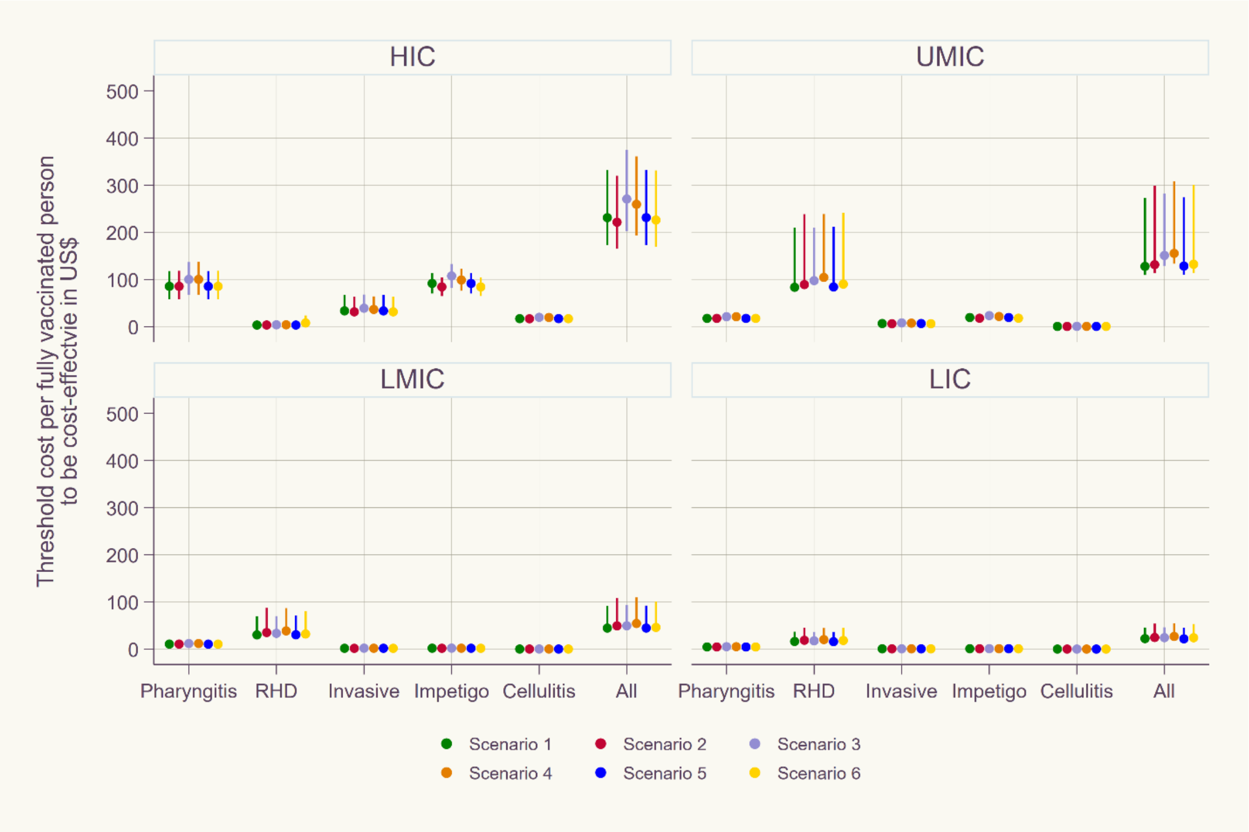

The lower bounds are based on the least favorable scenario: 20% wastage rate, lower bound of economic burden, and 3% discounting of health outcomes. The upper bounds are for the most favorable scenario: 5% wastage rate, upper bound of economic burden, and 0% discounting of health outcomes.

Supplementary Figure 4. Threshold cost per fully vaccinated person to be cost-effective by scenario: 60% reduction of the default efficacy rate scenario

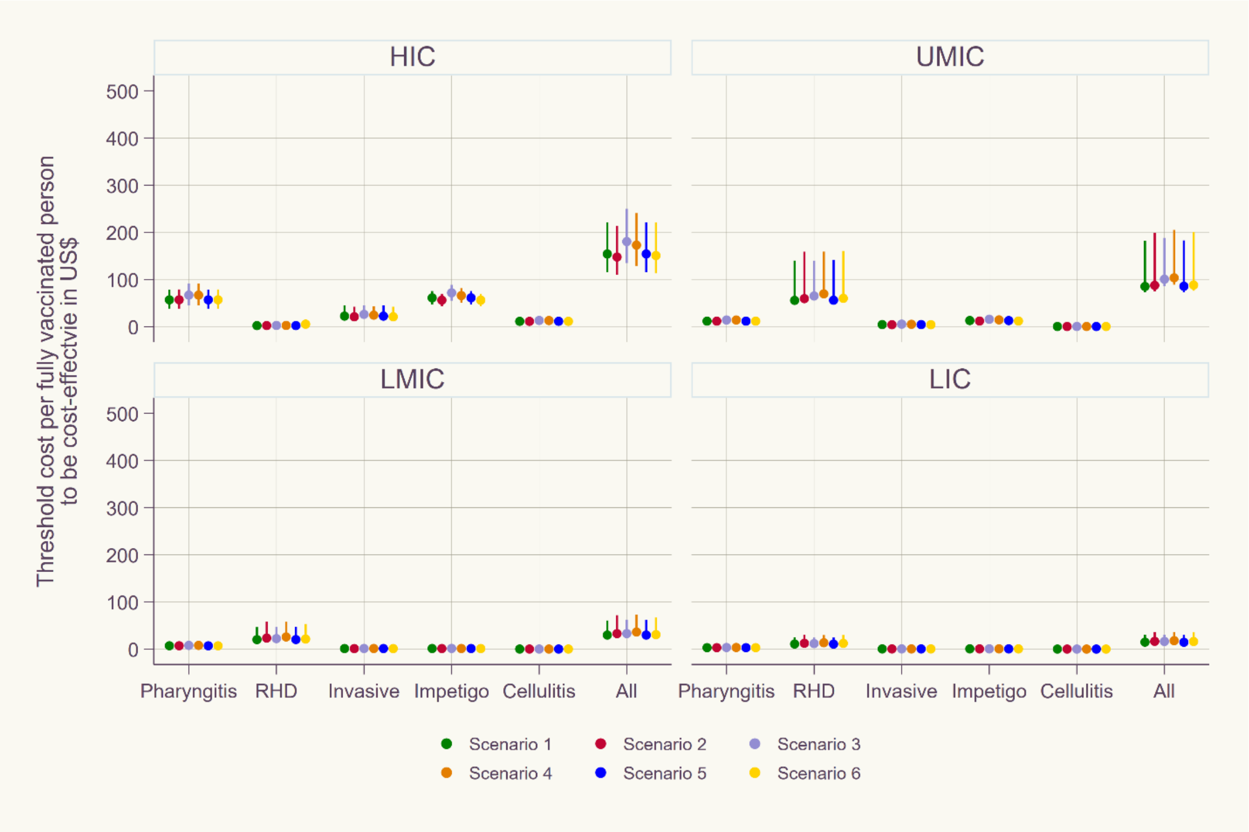

The lower bounds are based on the least favorable scenario: 20% wastage rate, lower bound of economic burden, and 3% discounting of health outcomes. The upper bounds are for the most favorable scenario: 5% wastage rate, upper bound of economic burden, and 0% discounting of health outcomes.

Supplementary Figure 5. Additional univariate analyses

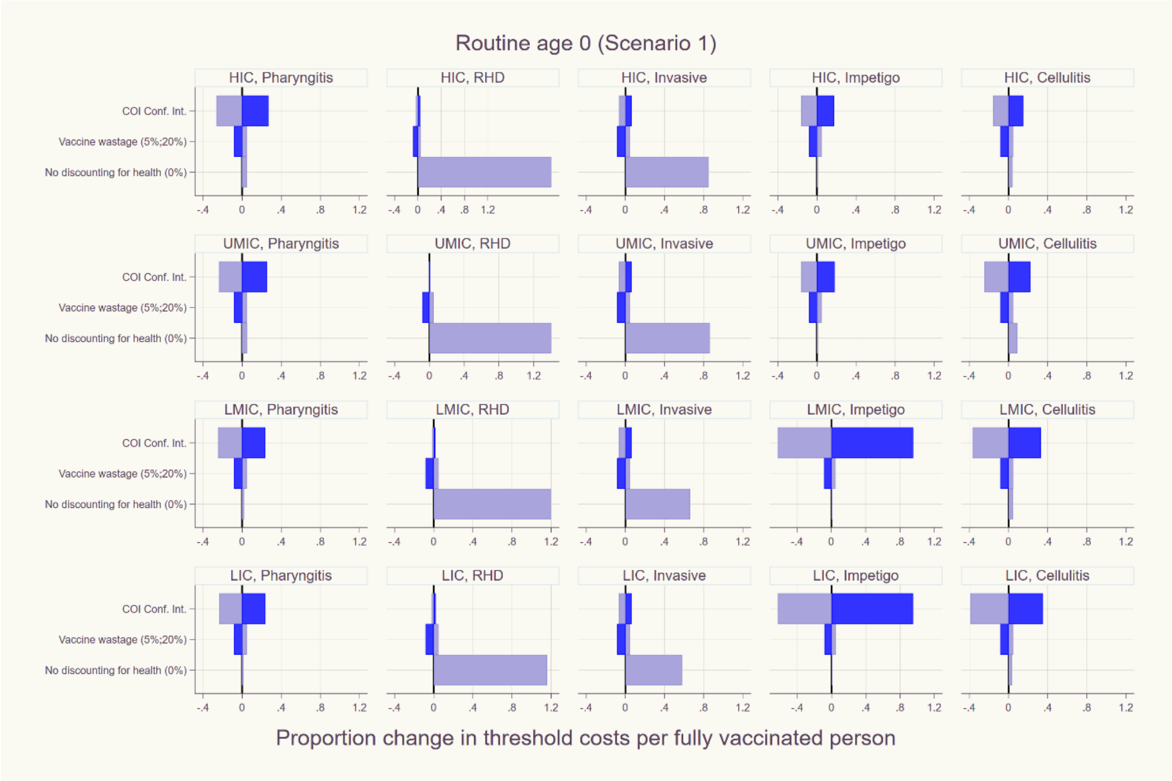

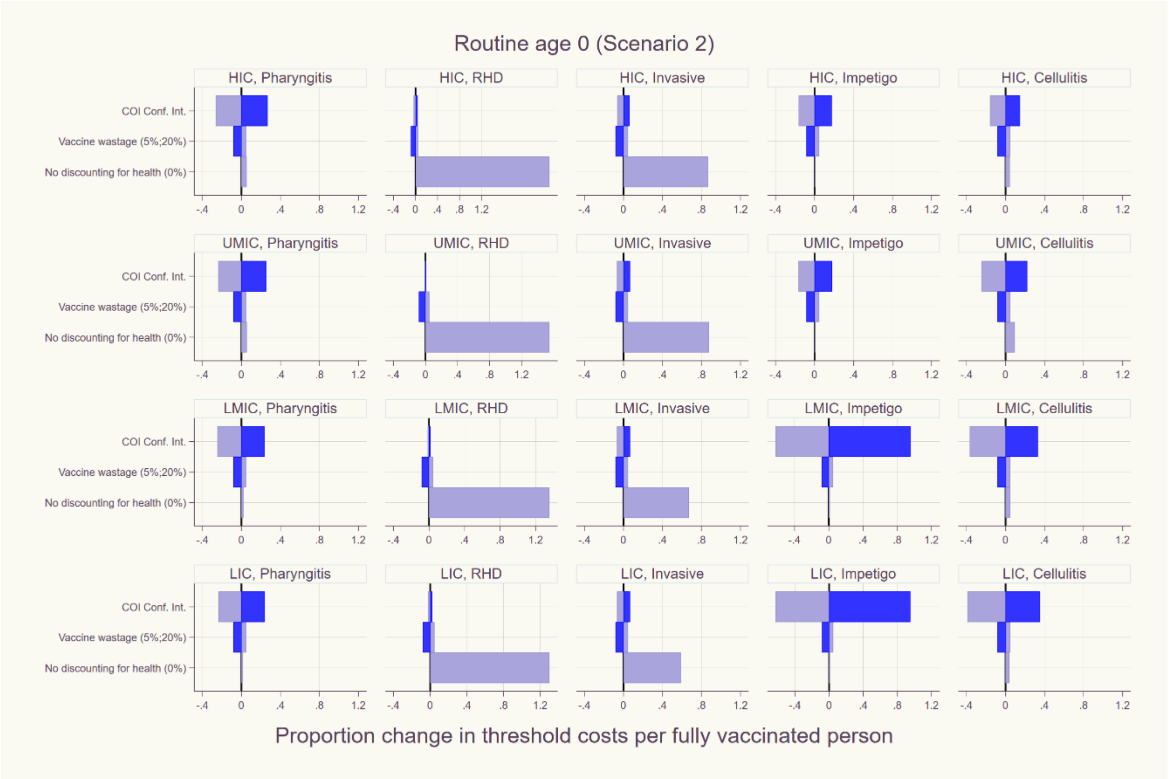

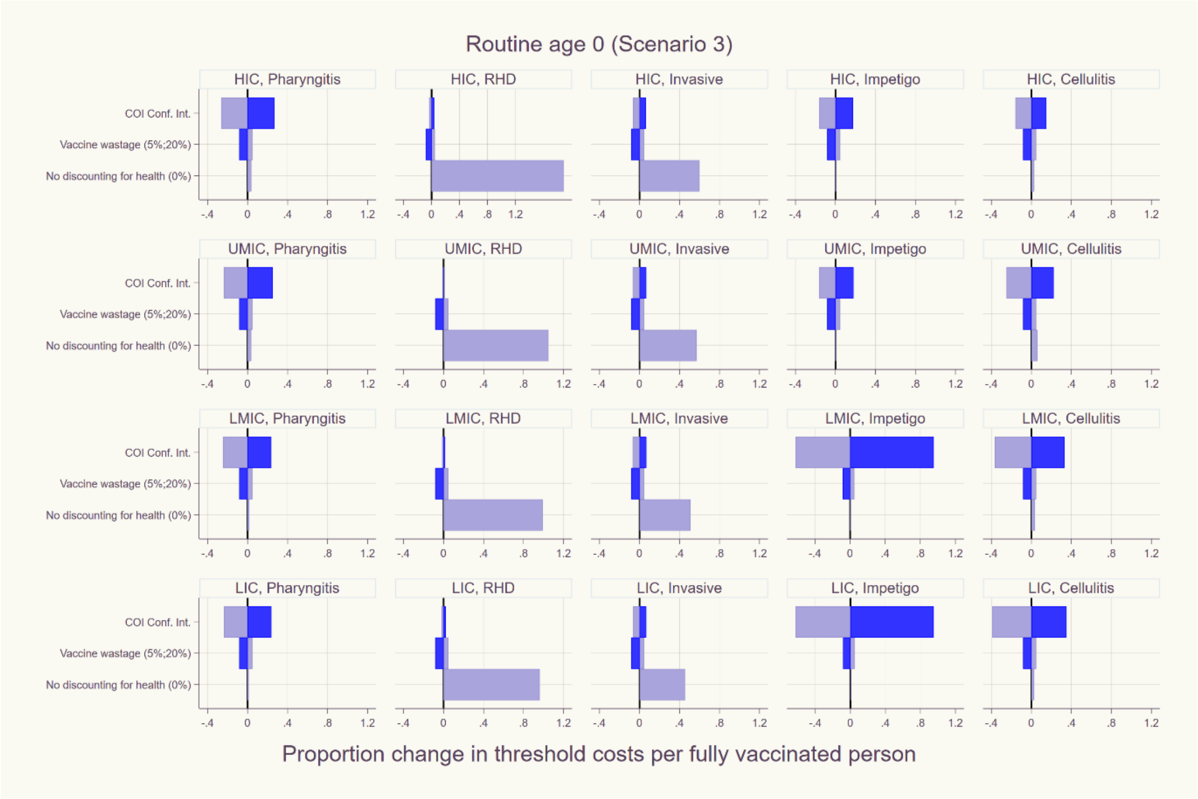

Routine age 0 (Scenario 3)
HIC, Pharyngitis
HIC, RHD
HIC, Invasive
HIC, Impetigo
HIC, Cellulitis
UMIC, Pharyngitis
UMIC, RHD
UMIC, Invasive
UMIC, Impetigo
UMIC, Cellulitis
LMIC, Pharyngitis
LMIC, RHD
LMIC, Invasive
LMIC, Impetigo
LMIC, Cellulitis
LIC, Pharyngitis
LIC, RHD
LIC, Invasive
LIC, Impetigo
LIC, Cellulitis
COI Conf. Int.
Vaccine wastage (5%;20%)
No discounting for health (0%)
Proportion change in threshold costs per fully vaccinated person

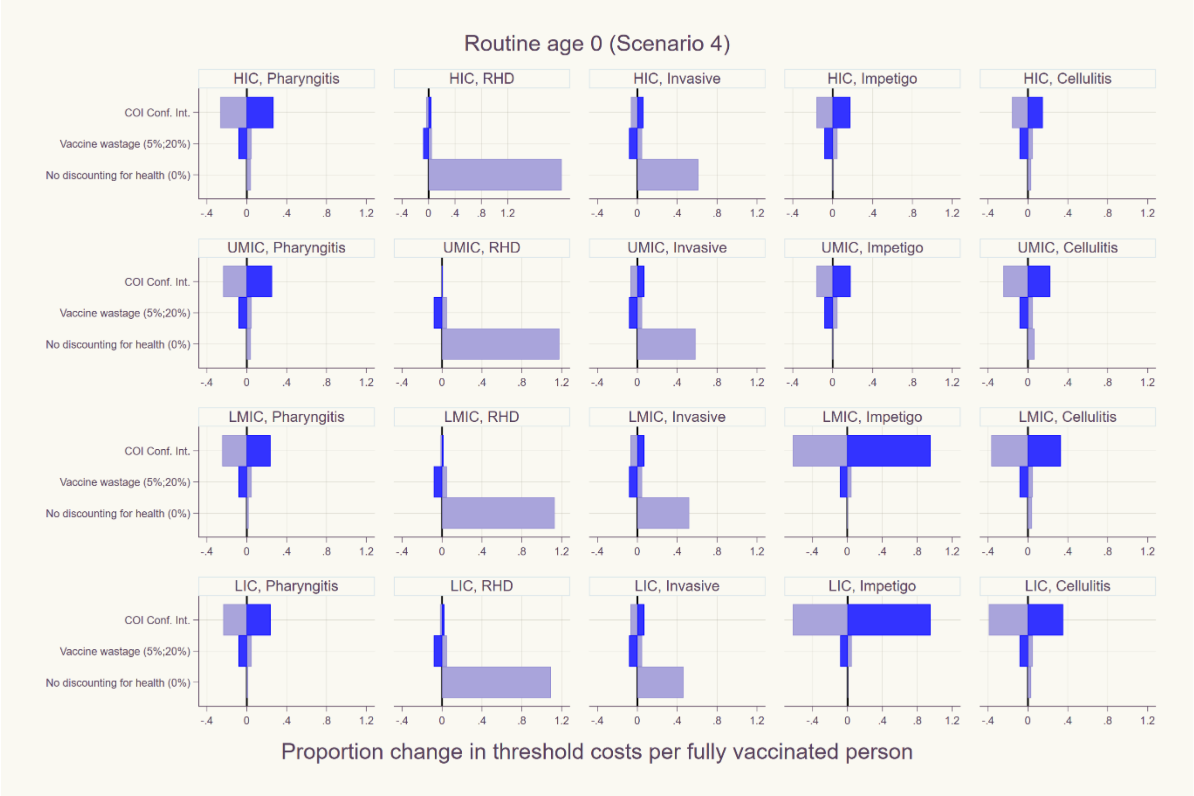

Routine age 0 (Scenario 4)
HIC, Pharyngitis
HIC, RHD
HIC, Invasive
HIC, Impetigo
HIC, Cellulitis
UMIC, Pharyngitis
UMIC, RHD
UMIC, Invasive
UMIC, Impetigo
UMIC, Cellulitis
LMIC, Pharyngitis
LMIC, RHD
LMIC, Invasive
LMIC, Impetigo
LMIC, Cellulitis
LIC, Pharyngitis
LIC, RHD
LIC, Invasive
LIC, Impetigo
LIC, Cellulitis
COI Conf. Int.
Vaccine wastage (5%;20%)
No discounting for health (0%)
Proportion change in threshold costs per fully vaccinated person

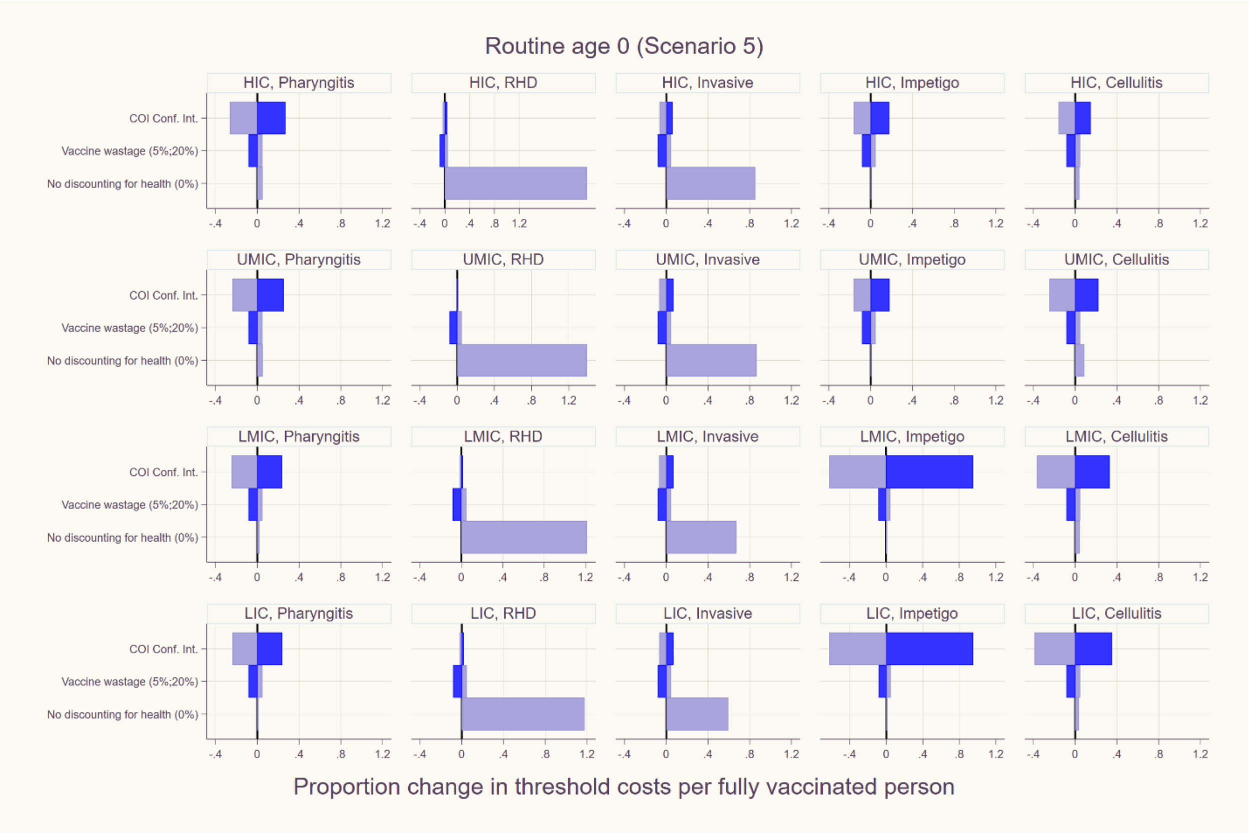
Routine age 0 (Scenario 5)
HIC, Pharyngitis
HIC, RHD
HIC, Invasive
HIC, Impetigo
HIC, Cellulitis
UMIC, Pharyngitis
UMIC, RHD
UMIC, Invasive
UMIC, Impetigo
UMIC, Cellulitis
LMIC, Pharyngitis
LMIC, RHD
LMIC, Invasive
LMIC, Impetigo
LMIC, Cellulitis
LIC, Pharyngitis
LIC, RHD
LIC, Invasive
LIC, Impetigo
LIC, Cellulitis
COI Conf. Int.
Vaccine wastage (5%;20%)
No discounting for health (0%)
-.4
0
.4
.8
1.2
Proportion change in threshold costs per fully vaccinated person

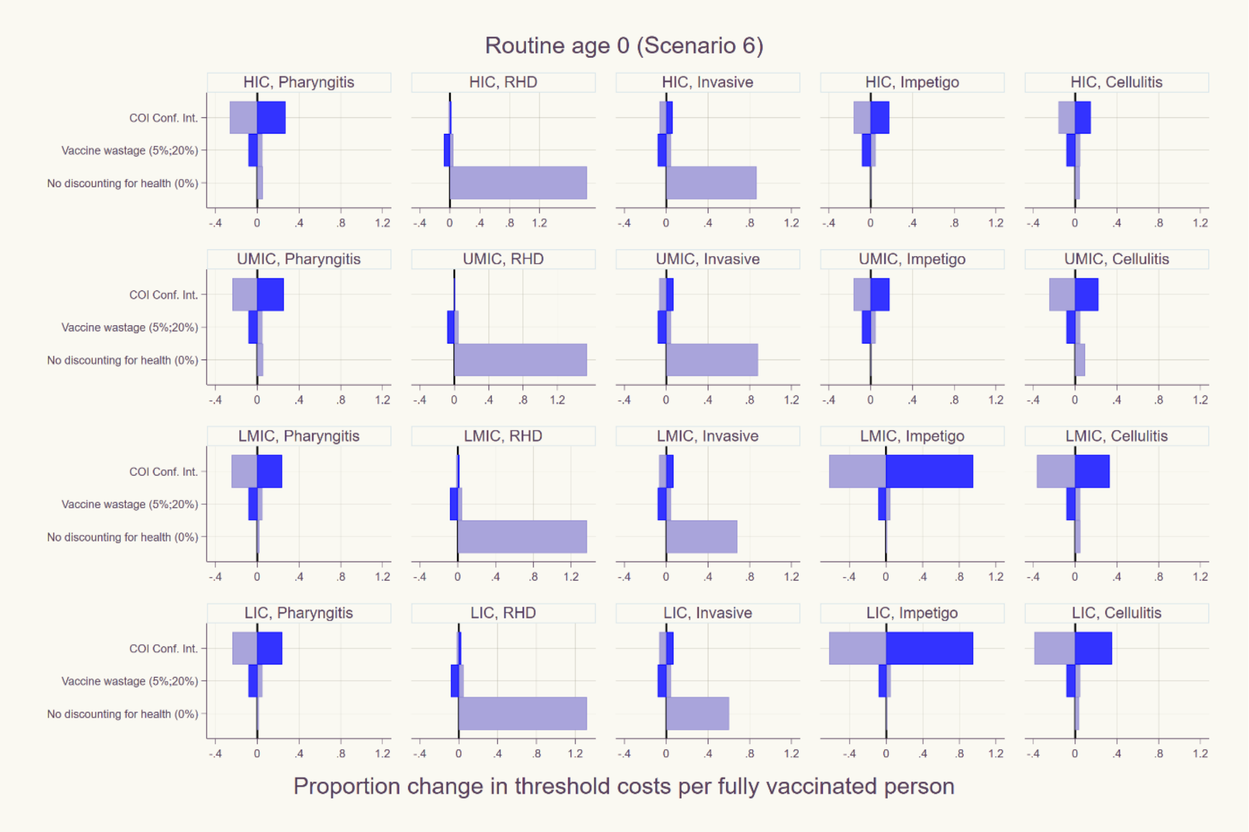
Routine age 0 (Scenario 6)
HIC, Pharyngitis
HIC, RHD
HIC, Invasive
HIC, Impetigo
HIC, Cellulitis
UMIC, Pharyngitis
UMIC, RHD
UMIC, Invasive
UMIC, Impetigo
UMIC, Cellulitis
LMIC, Pharyngitis
LMIC, RHD
LMIC, Invasive
LMIC, Impetigo
LMIC, Cellulitis
LIC, Pharyngitis
LIC, RHD
LIC, Invasive
LIC, Impetigo
LIC, Cellulitis
COI Conf. Int.
Vaccine wastage (5%;20%)
No discounting for health (0%)
-.4
0
.4
.8
1.2
Proportion change in threshold costs per fully vaccinated person

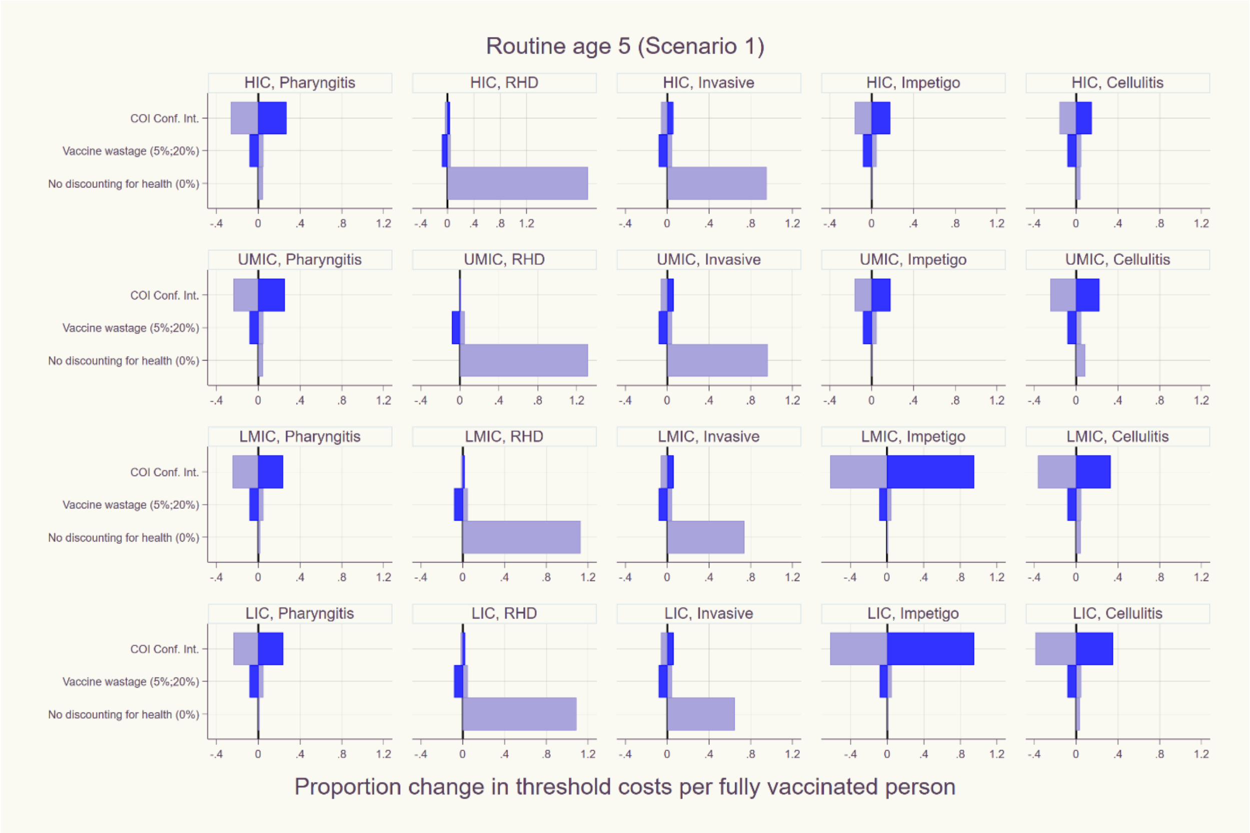
Routine age 5 (Scenario 1)
HIC, Pharyngitis
HIC, RHD
HIC, Invasive
HIC, Impetigo
HIC, Cellulitis
UMIC, Pharyngitis
UMIC, RHD
UMIC, Invasive
UMIC, Impetigo
UMIC, Cellulitis
LMIC, Pharyngitis
LMIC, RHD
LMIC, Invasive
LMIC, Impetigo
LMIC, Cellulitis
LIC, Pharyngitis
LIC, RHD
LIC, Invasive
LIC, Impetigo
LIC, Cellulitis
COI Conf. Int.
Vaccine wastage (5%;20%)
No discounting for health (0%)
-.4
0
.4
.8
1.2
Proportion change in threshold costs per fully vaccinated person

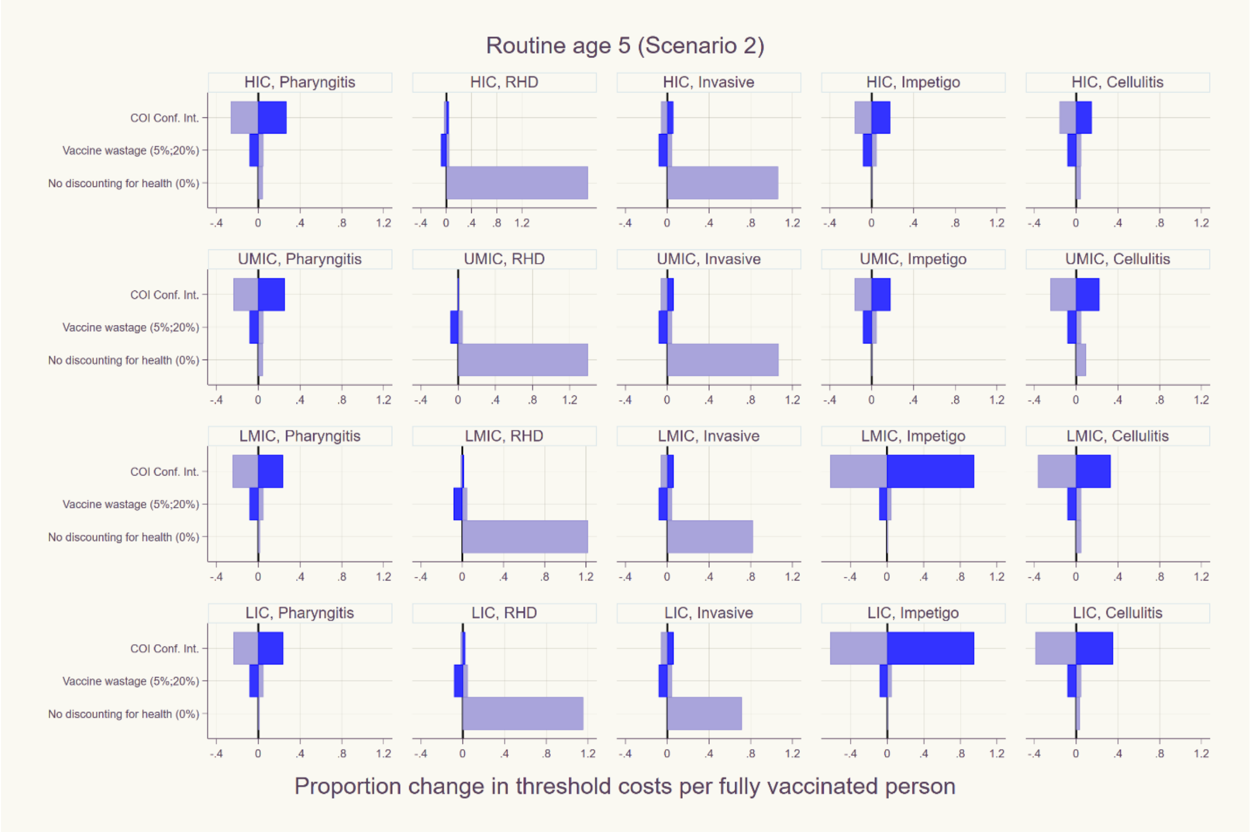
Routine age 5 (Scenario 2)
HIC, Pharyngitis
HIC, RHD
HIC, Invasive
HIC, Impetigo
HIC, Cellulitis
UMIC, Pharyngitis
UMIC, RHD
UMIC, Invasive
UMIC, Impetigo
UMIC, Cellulitis
LMIC, Pharyngitis
LMIC, RHD
LMIC, Invasive
LMIC, Impetigo
LMIC, Cellulitis
LIC, Pharyngitis
LIC, RHD
LIC, Invasive
LIC, Impetigo
LIC, Cellulitis
COI Conf. Int.
Vaccine wastage (5%;20%)
No discounting for health (0%)
-.4
0
.4
.8
1.2
Proportion change in threshold costs per fully vaccinated person

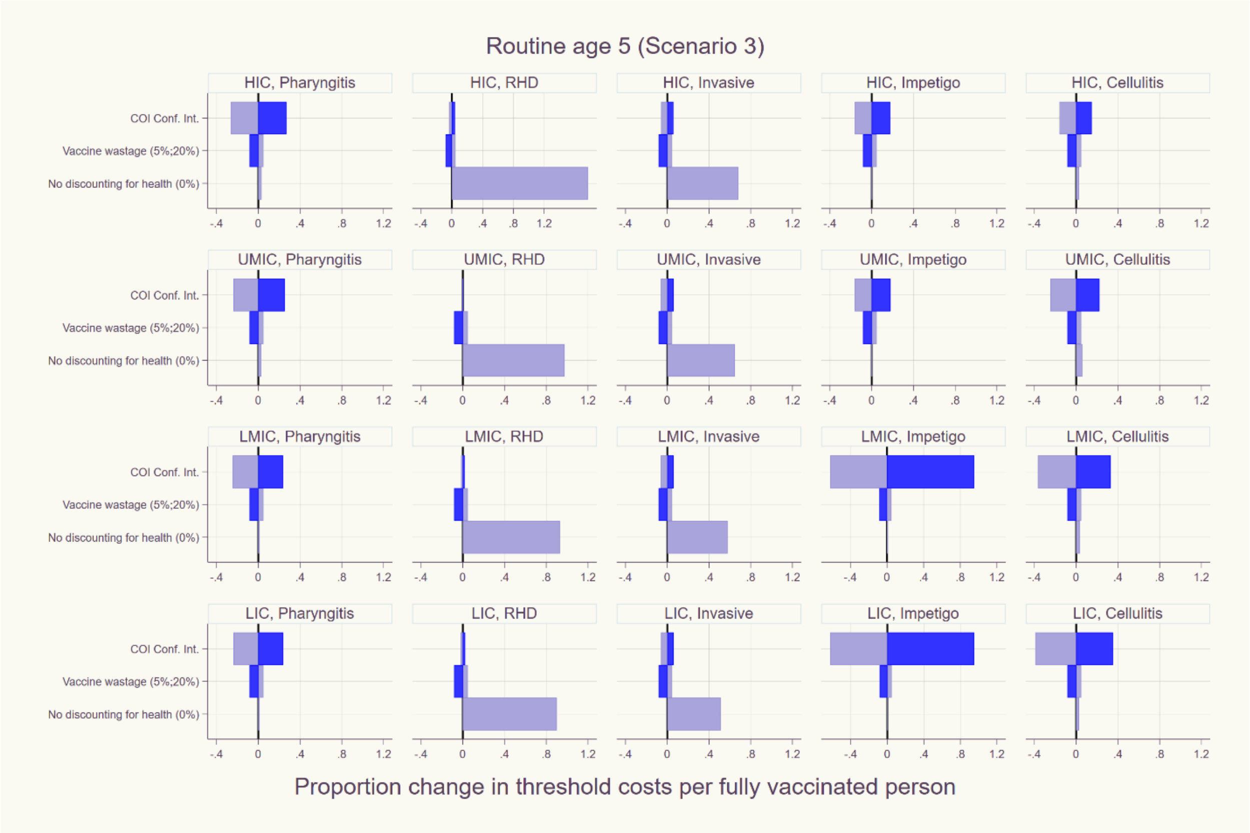
Routine age 5 (Scenario 3)
HIC, Pharyngitis
HIC, RHD
HIC, Invasive
HIC, Impetigo
HIC, Cellulitis
UMIC, Pharyngitis
UMIC, RHD
UMIC, Invasive
UMIC, Impetigo
UMIC, Cellulitis
LMIC, Pharyngitis
LMIC, RHD
LMIC, Invasive
LMIC, Impetigo
LMIC, Cellulitis
LIC, Pharyngitis
LIC, RHD
LIC, Invasive
LIC, Impetigo
LIC, Cellulitis
COI Conf. Int.
Vaccine wastage (5%;20%)
No discounting for health (0%)
-.4
0
.4
.8
1.2
Proportion change in threshold costs per fully vaccinated person

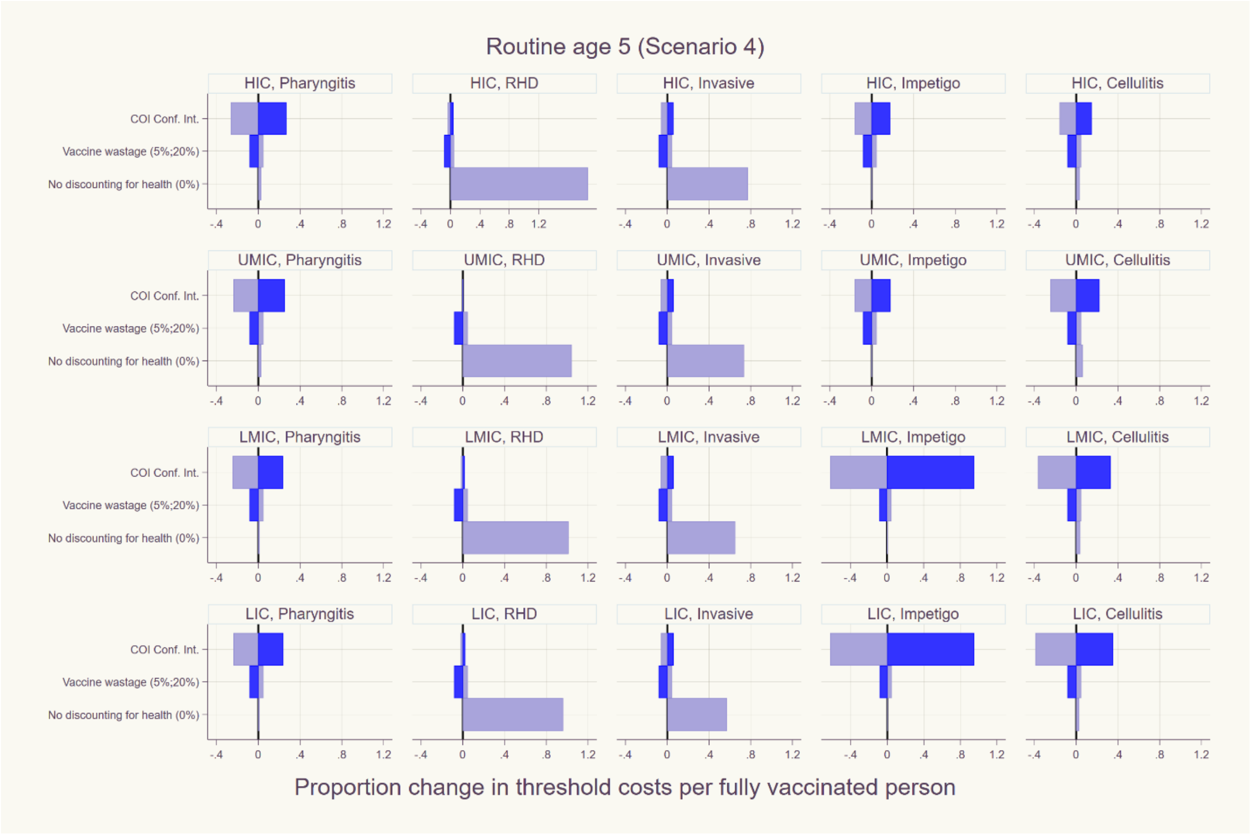
Routine age 5 (Scenario 4)
HIC, Pharyngitis
HIC, RHD
HIC, Invasive
HIC, Impetigo
HIC, Cellulitis
UMIC, Pharyngitis
UMIC, RHD
UMIC, Invasive
UMIC, Impetigo
UMIC, Cellulitis
LMIC, Pharyngitis
LMIC, RHD
LMIC, Invasive
LMIC, Impetigo
LMIC, Cellulitis
LIC, Pharyngitis
LIC, RHD
LIC, Invasive
LIC, Impetigo
LIC, Cellulitis
COI Conf. Int.
Vaccine wastage (5%;20%)
No discounting for health (0%)
-.4
0
.4
.8
1.2
Proportion change in threshold costs per fully vaccinated person

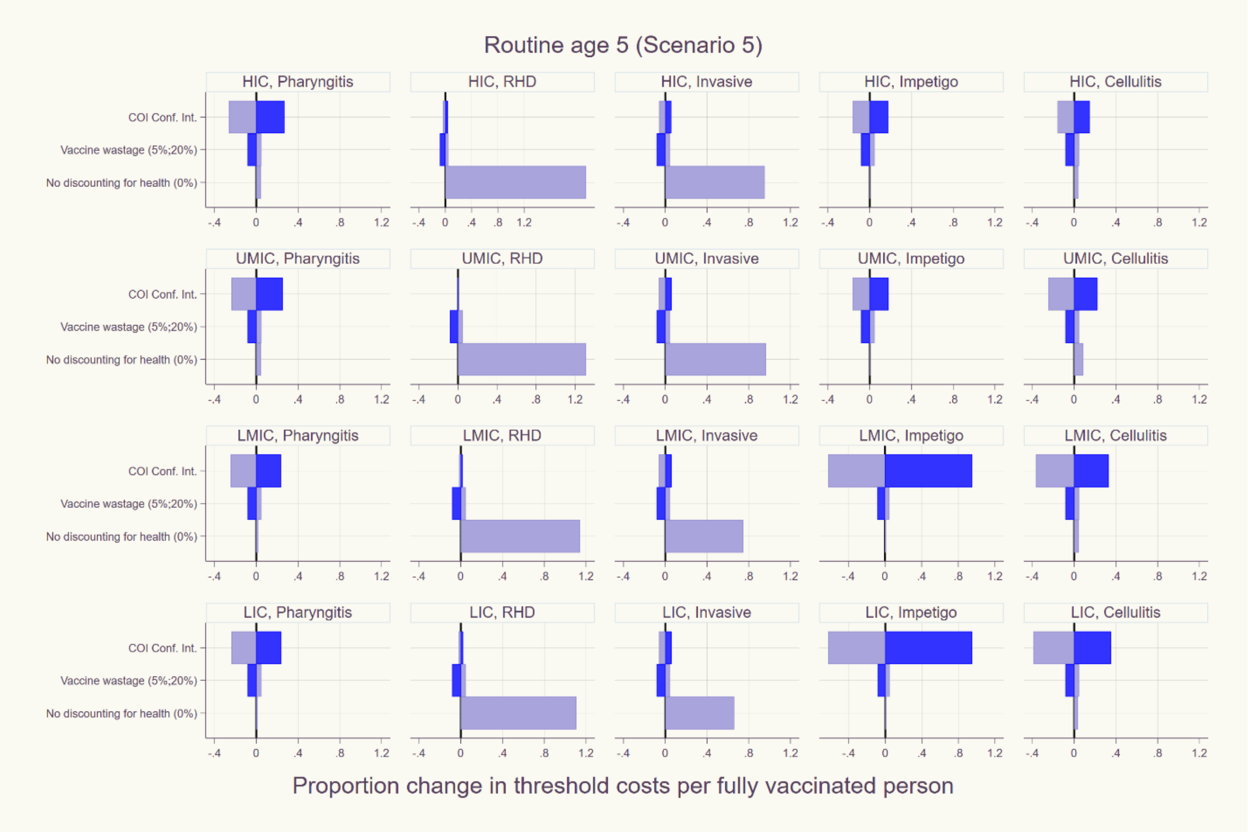
Routine age 5 (Scenario 5)
HIC, Pharyngitis
HIC, RHD
HIC, Invasive
HIC, Impetigo
HIC, Cellulitis
UMIC, Pharyngitis
UMIC, RHD
UMIC, Invasive
UMIC, Impetigo
UMIC, Cellulitis
LMIC, Pharyngitis
LMIC, RHD
LMIC, Invasive
LMIC, Impetigo
LMIC, Cellulitis
LIC, Pharyngitis
LIC, RHD
LIC, Invasive
LIC, Impetigo
LIC, Cellulitis
COI Conf. Int.
Vaccine wastage (5%;20%)
No discounting for health (0%)
-.4
0
.4
.8
1.2
Proportion change in threshold costs per fully vaccinated person

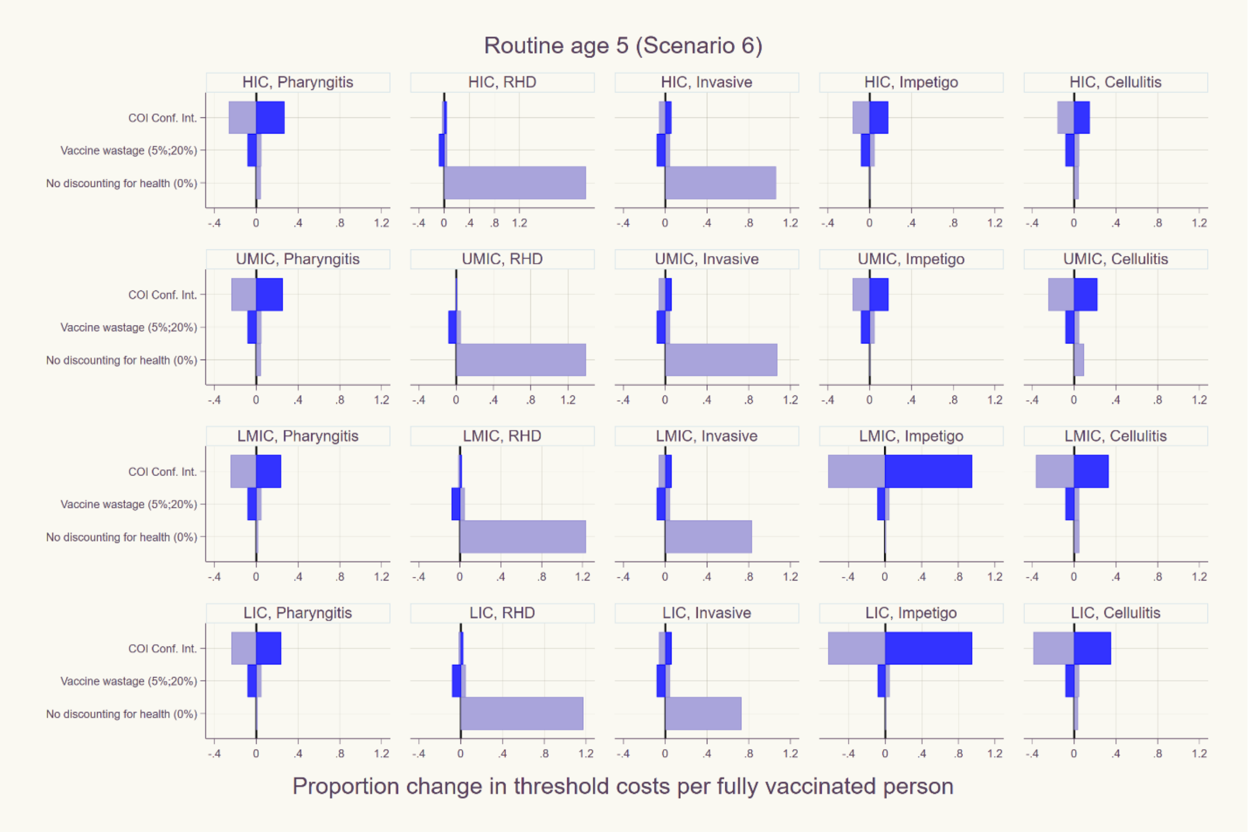
Routine age 5 (Scenario 6)
HIC, Pharyngitis
HIC, RHD
HIC, Invasive
HIC, Impetigo
HIC, Cellulitis
UMIC, Pharyngitis
UMIC, RHD
UMIC, Invasive
UMIC, Impetigo
UMIC, Cellulitis
LMIC, Pharyngitis
LMIC, RHD
LMIC, Invasive
LMIC, Impetigo
LMIC, Cellulitis
LIC, Pharyngitis
LIC, RHD
LIC, Invasive
LIC, Impetigo
LIC, Cellulitis
COI Conf. Int.
Vaccine wastage (5%;20%)
No discounting for health (0%)
-.4
0
.4
.8
1.2
Proportion change in threshold costs per fully vaccinated person

Supplementary Figure 6. Additional multivariate analyses

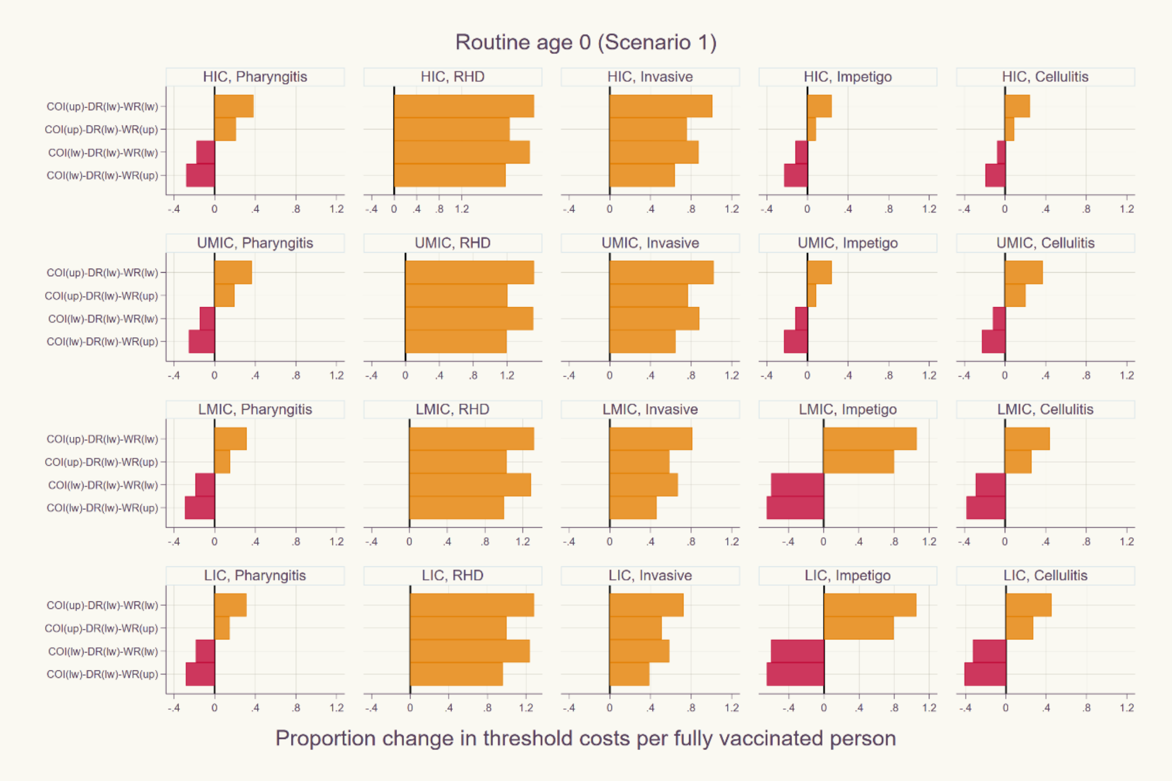

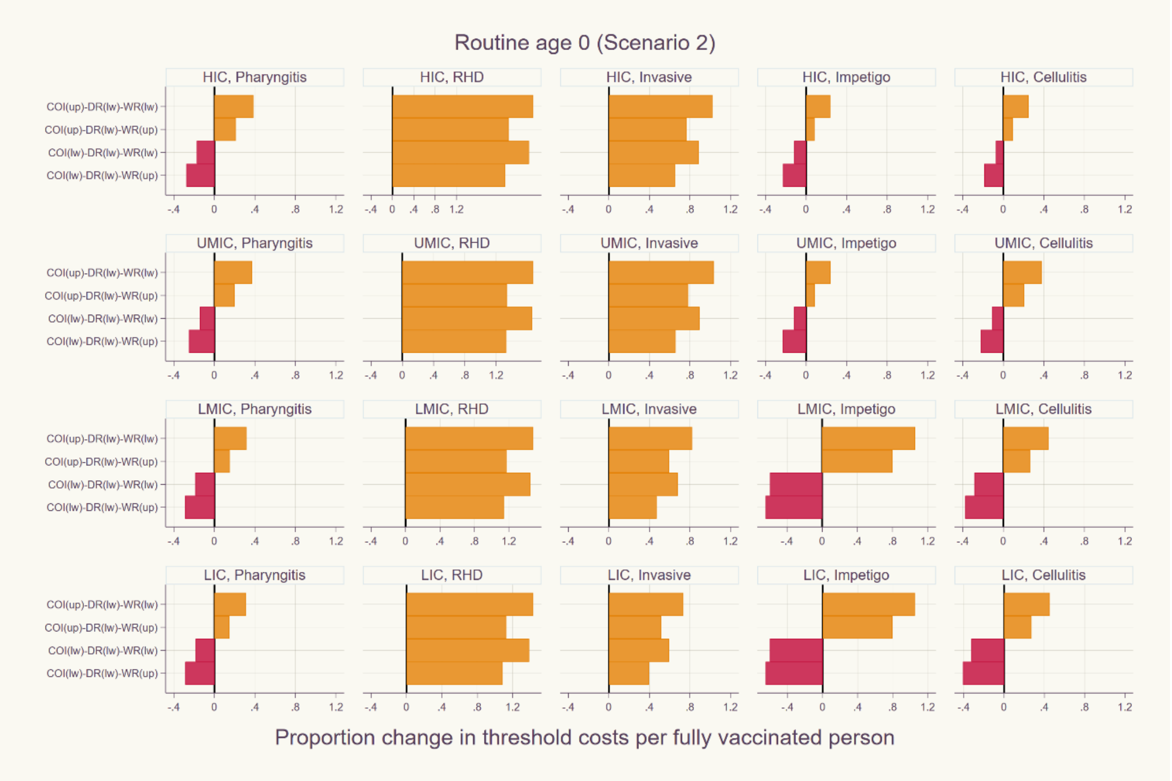

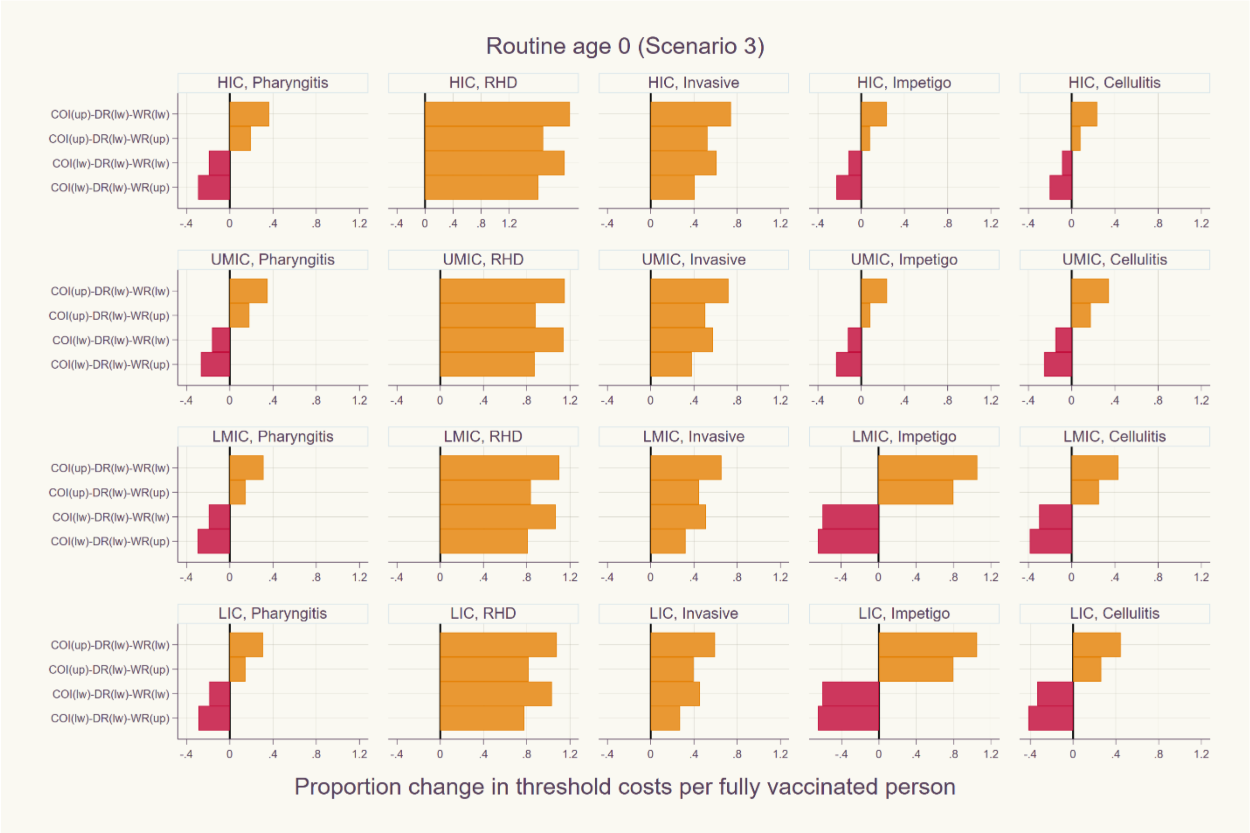
Routine age 0 (Scenario 3)
HIC, Pharyngitis
HIC, RHD
HIC, Invasive
HIC, Impetigo
HIC, Cellulitis
UMIC, Pharyngitis
UMIC, RHD
UMIC, Invasive
UMIC, Impetigo
UMIC, Cellulitis
LMIC, Pharyngitis
LMIC, RHD
LMIC, Invasive
LMIC, Impetigo
LMIC, Cellulitis
LIC, Pharyngitis
LIC, RHD
LIC, Invasive
LIC, Impetigo
LIC, Cellulitis
COI(up)-DR(lw)-WR(lw)
COI(up)-DR(lw)-WR(up)
COI(lw)-DR(lw)-WR(lw)
COI(lw)-DR(lw)-WR(up)
-.4
0
.4
.8
1.2
Proportion change in threshold costs per fully vaccinated person

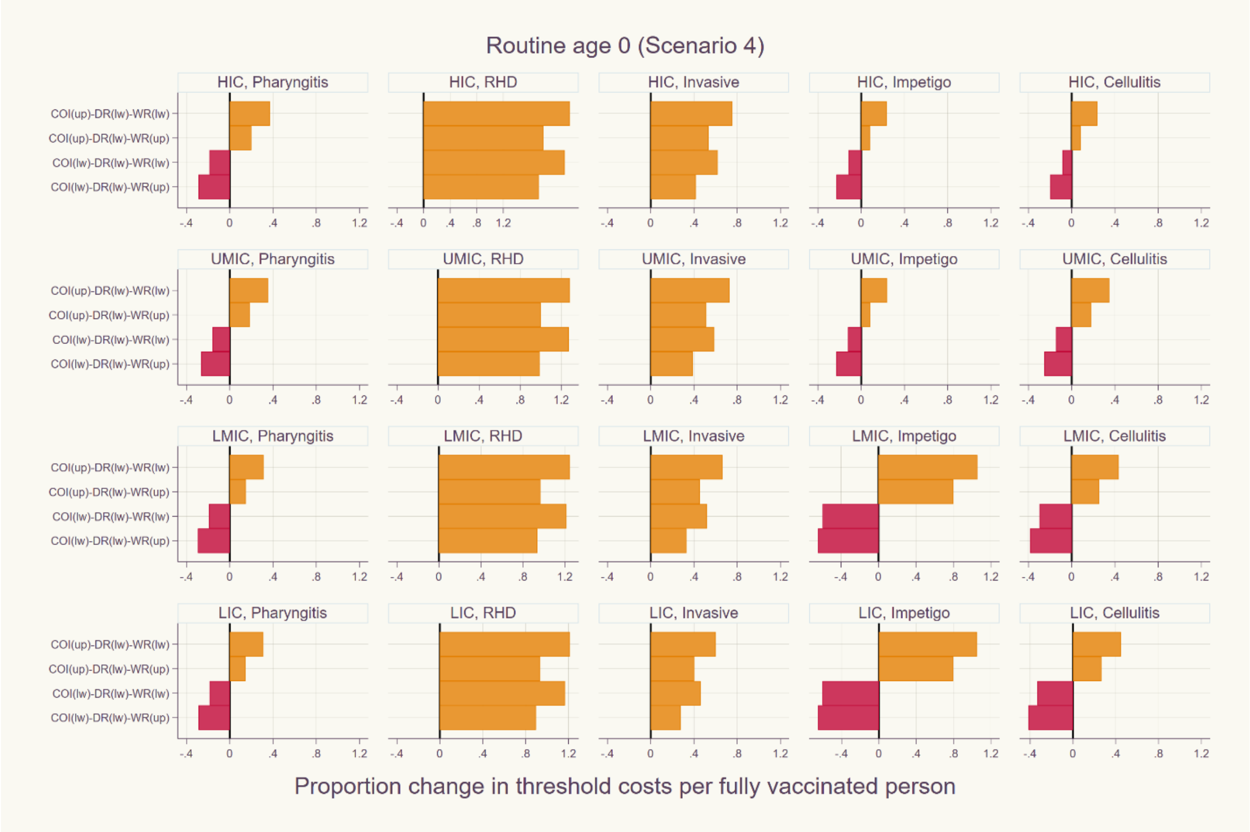
Routine age 0 (Scenario 4)
HIC, Pharyngitis
HIC, RHD
HIC, Invasive
HIC, Impetigo
HIC, Cellulitis
UMIC, Pharyngitis
UMIC, RHD
UMIC, Invasive
UMIC, Impetigo
UMIC, Cellulitis
LMIC, Pharyngitis
LMIC, RHD
LMIC, Invasive
LMIC, Impetigo
LMIC, Cellulitis
LIC, Pharyngitis
LIC, RHD
LIC, Invasive
LIC, Impetigo
LIC, Cellulitis
COI(up)-DR(lw)-WR(lw)
COI(up)-DR(lw)-WR(up)
COI(lw)-DR(lw)-WR(lw)
COI(lw)-DR(lw)-WR(up)
-.4
0
.4
.8
1.2
Proportion change in threshold costs per fully vaccinated person

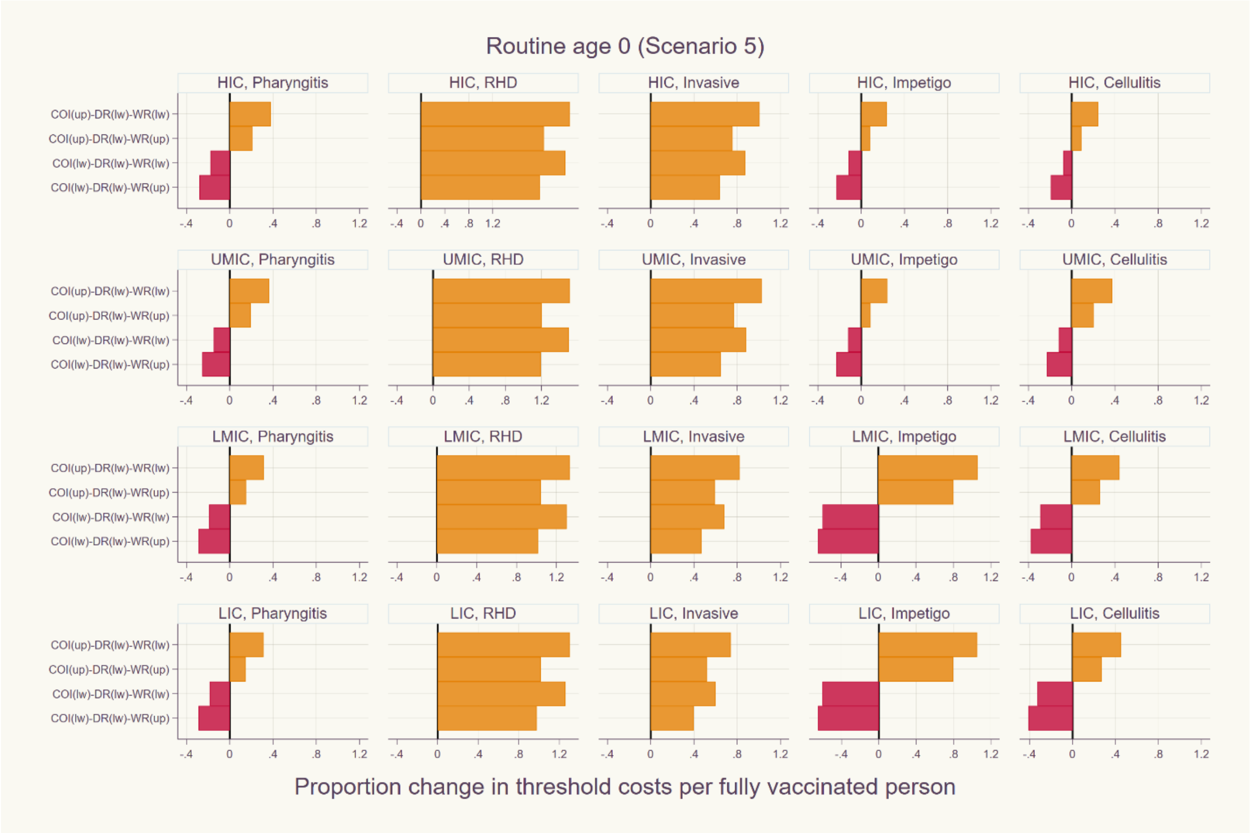
Routine age 0 (Scenario 5)
HIC, Pharyngitis
HIC, RHD
HIC, Invasive
HIC, Impetigo
HIC, Cellulitis
UMIC, Pharyngitis
UMIC, RHD
UMIC, Invasive
UMIC, Impetigo
UMIC, Cellulitis
LMIC, Pharyngitis
LMIC, RHD
LMIC, Invasive
LMIC, Impetigo
LMIC, Cellulitis
LIC, Pharyngitis
LIC, RHD
LIC, Invasive
LIC, Impetigo
LIC, Cellulitis
COI(up)-DR(lw)-WR(lw)
COI(up)-DR(lw)-WR(up)
COI(lw)-DR(lw)-WR(lw)
COI(lw)-DR(lw)-WR(up)
-.4
0
.4
.8
1.2
Proportion change in threshold costs per fully vaccinated person

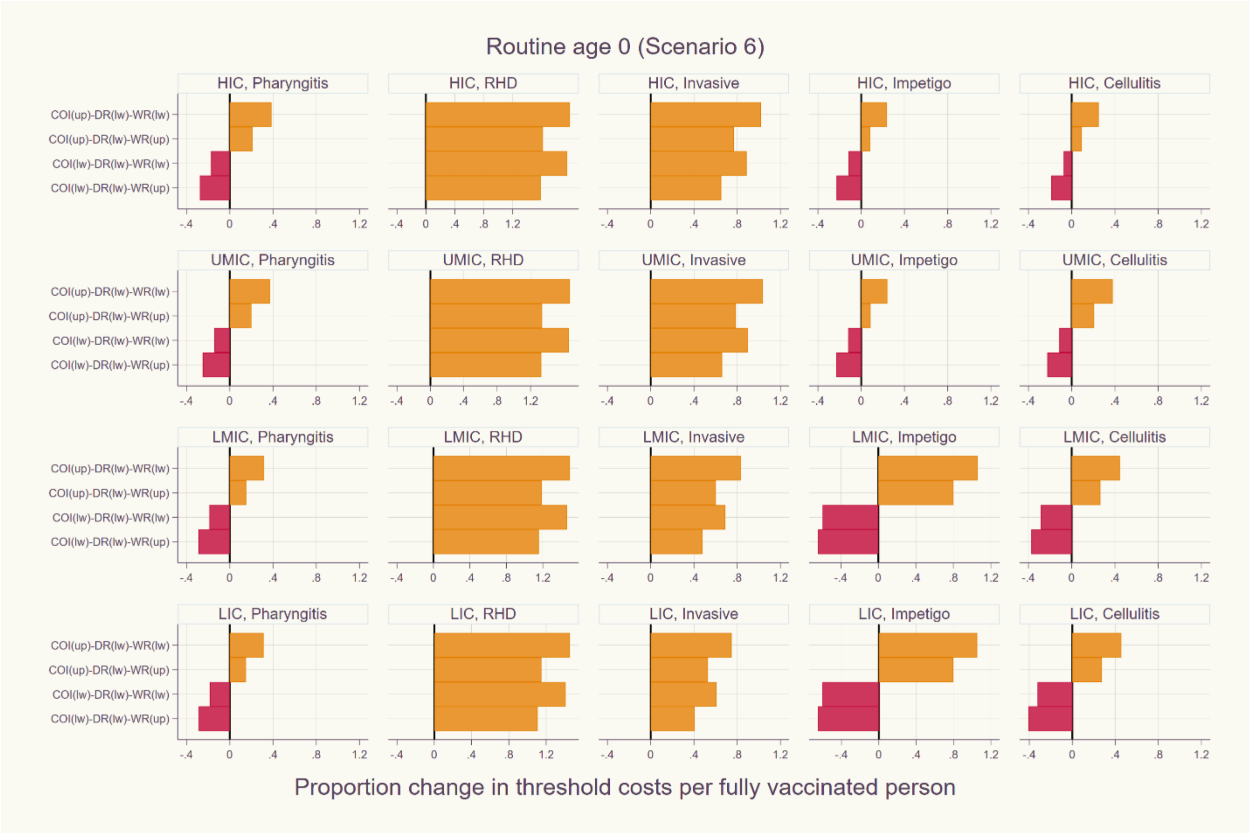
Routine age 0 (Scenario 6)
HIC, Pharyngitis
HIC, RHD
HIC, Invasive
HIC, Impetigo
HIC, Cellulitis
UMIC, Pharyngitis
UMIC, RHD
UMIC, Invasive
UMIC, Impetigo
UMIC, Cellulitis
LMIC, Pharyngitis
LMIC, RHD
LMIC, Invasive
LMIC, Impetigo
LMIC, Cellulitis
LIC, Pharyngitis
LIC, RHD
LIC, Invasive
LIC, Impetigo
LIC, Cellulitis
COI(up)-DR(lw)-WR(lw)
COI(up)-DR(lw)-WR(up)
COI(lw)-DR(lw)-WR(lw)
COI(lw)-DR(lw)-WR(up)
-.4
0
.4
.8
1.2
Proportion change in threshold costs per fully vaccinated person

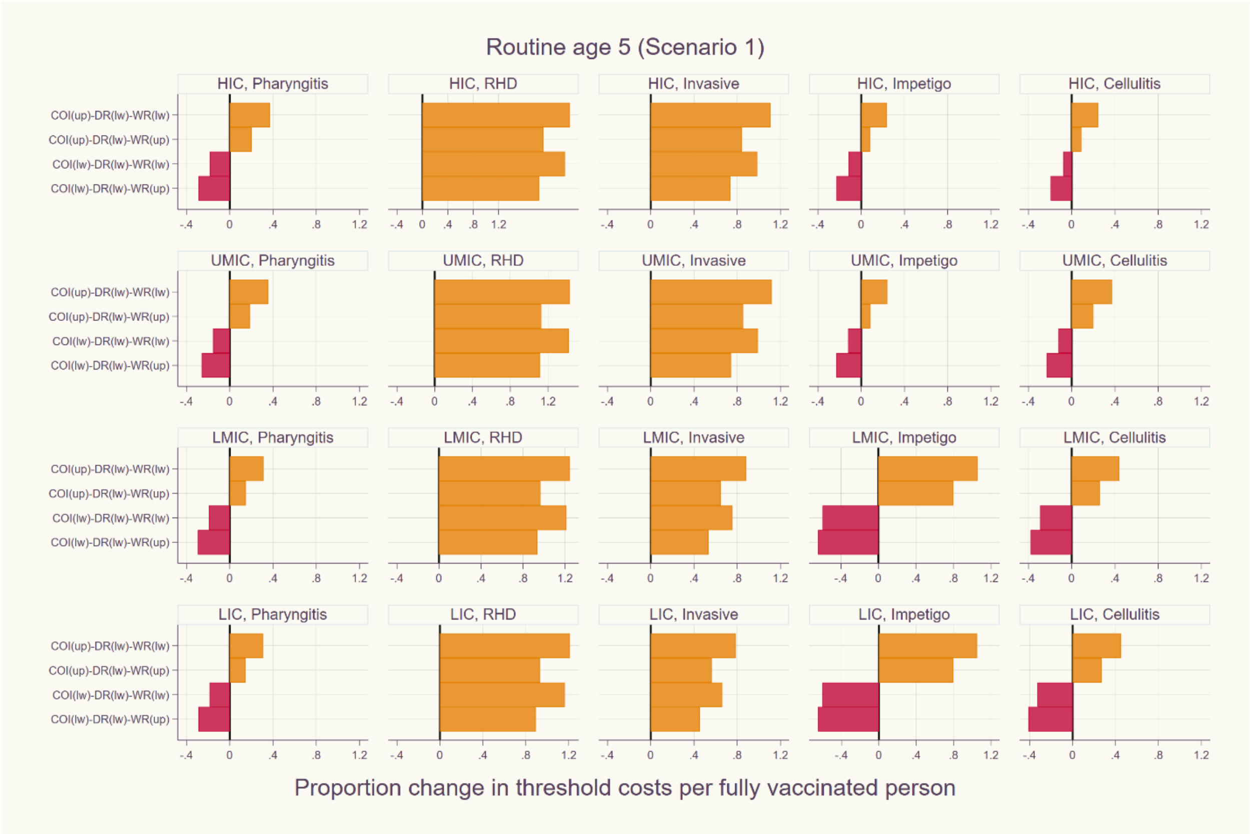
Routine age 5 (Scenario 1)
HIC, Pharyngitis
HIC, RHD
HIC, Invasive
HIC, Impetigo
HIC, Cellulitis
UMIC, Pharyngitis
UMIC, RHD
UMIC, Invasive
UMIC, Impetigo
UMIC, Cellulitis
LMIC, Pharyngitis
LMIC, RHD
LMIC, Invasive
LMIC, Impetigo
LMIC, Cellulitis
LIC, Pharyngitis
LIC, RHD
LIC, Invasive
LIC, Impetigo
LIC, Cellulitis
COI(up)-DR(lw)-WR(lw)
COI(up)-DR(lw)-WR(up)
COI(lw)-DR(lw)-WR(lw)
COI(lw)-DR(lw)-WR(up)
Proportion change in threshold costs per fully vaccinated person

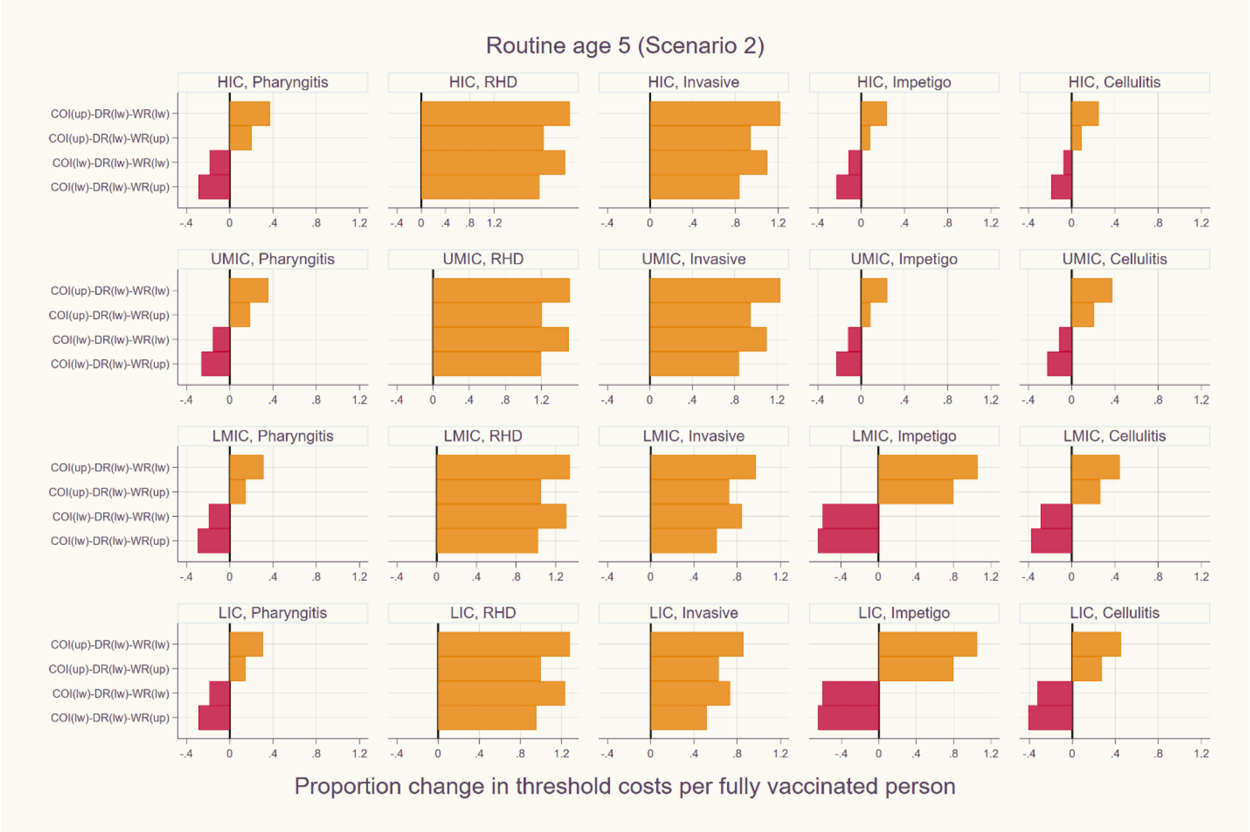
Routine age 5 (Scenario 2)
HIC, Pharyngitis
HIC, RHD
HIC, Invasive
HIC, Impetigo
HIC, Cellulitis
UMIC, Pharyngitis
UMIC, RHD
UMIC, Invasive
UMIC, Impetigo
UMIC, Cellulitis
LMIC, Pharyngitis
LMIC, RHD
LMIC, Invasive
LMIC, Impetigo
LMIC, Cellulitis
LIC, Pharyngitis
LIC, RHD
LIC, Invasive
LIC, Impetigo
LIC, Cellulitis
COI(up)-DR(lw)-WR(lw)
COI(up)-DR(lw)-WR(up)
COI(lw)-DR(lw)-WR(lw)
COI(lw)-DR(lw)-WR(up)
Proportion change in threshold costs per fully vaccinated person

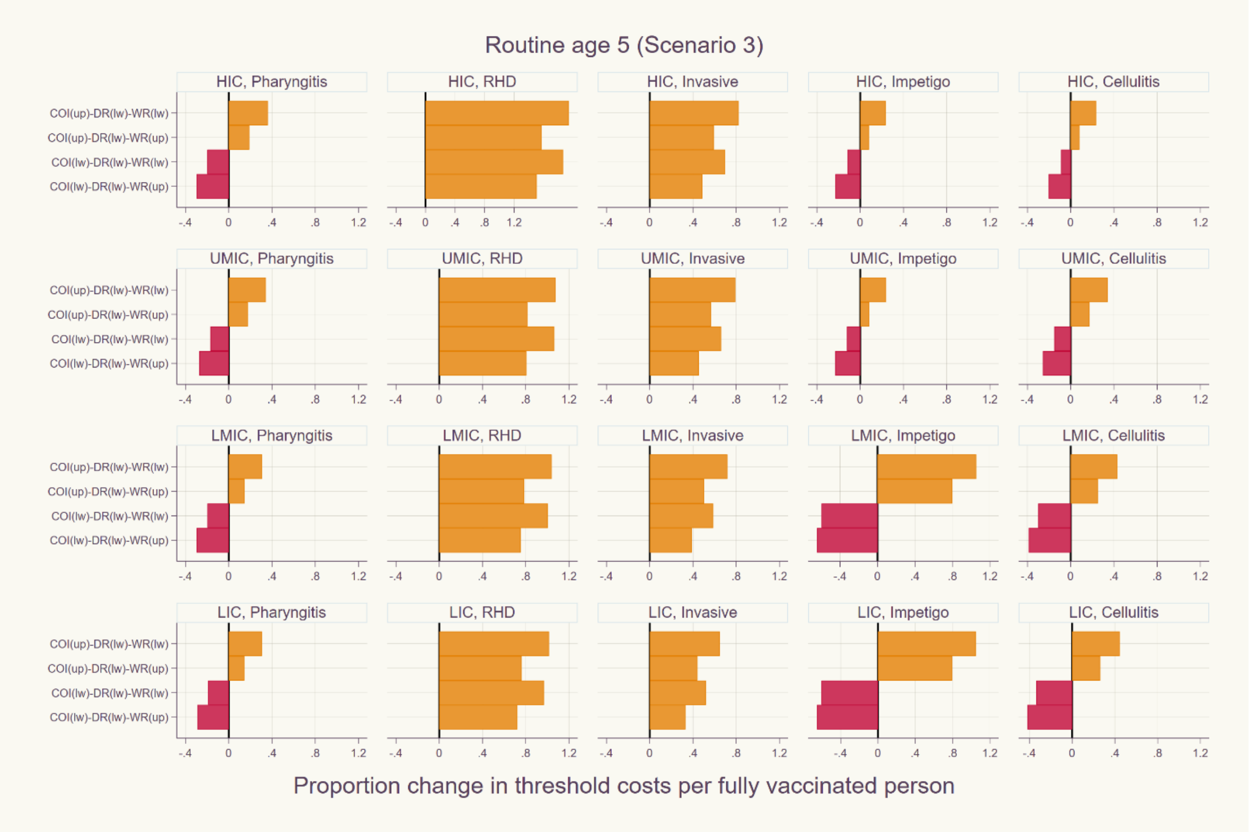
Routine age 5 (Scenario 3)
HIC, Pharyngitis
HIC, RHD
HIC, Invasive
HIC, Impetigo
HIC, Cellulitis
UMIC, Pharyngitis
UMIC, RHD
UMIC, Invasive
UMIC, Impetigo
UMIC, Cellulitis
LMIC, Pharyngitis
LMIC, RHD
LMIC, Invasive
LMIC, Impetigo
LMIC, Cellulitis
LIC, Pharyngitis
LIC, RHD
LIC, Invasive
LIC, Impetigo
LIC, Cellulitis
COI(up)-DR(lw)-WR(lw)
COI(up)-DR(lw)-WR(up)
COI(lw)-DR(lw)-WR(lw)
COI(lw)-DR(lw)-WR(up)
-.4
0
.4
.8
1.2
Proportion change in threshold costs per fully vaccinated person

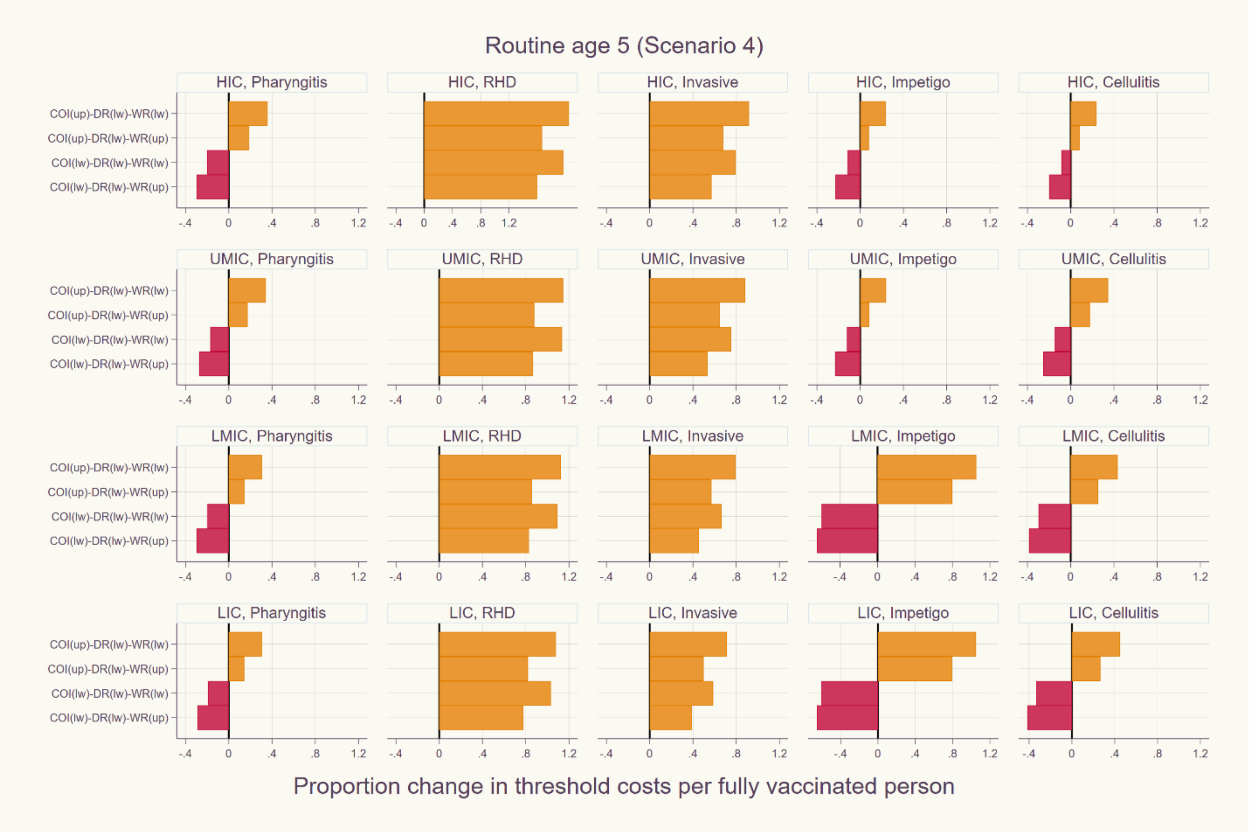
Routine age 5 (Scenario 4)
HIC, Pharyngitis
HIC, RHD
HIC, Invasive
HIC, Impetigo
HIC, Cellulitis
UMIC, Pharyngitis
UMIC, RHD
UMIC, Invasive
UMIC, Impetigo
UMIC, Cellulitis
LMIC, Pharyngitis
LMIC, RHD
LMIC, Invasive
LMIC, Impetigo
LMIC, Cellulitis
LIC, Pharyngitis
LIC, RHD
LIC, Invasive
LIC, Impetigo
LIC, Cellulitis
COI(up)-DR(lw)-WR(lw)
COI(up)-DR(lw)-WR(up)
COI(lw)-DR(lw)-WR(lw)
COI(lw)-DR(lw)-WR(up)
-.4
0
.4
.8
1.2
Proportion change in threshold costs per fully vaccinated person

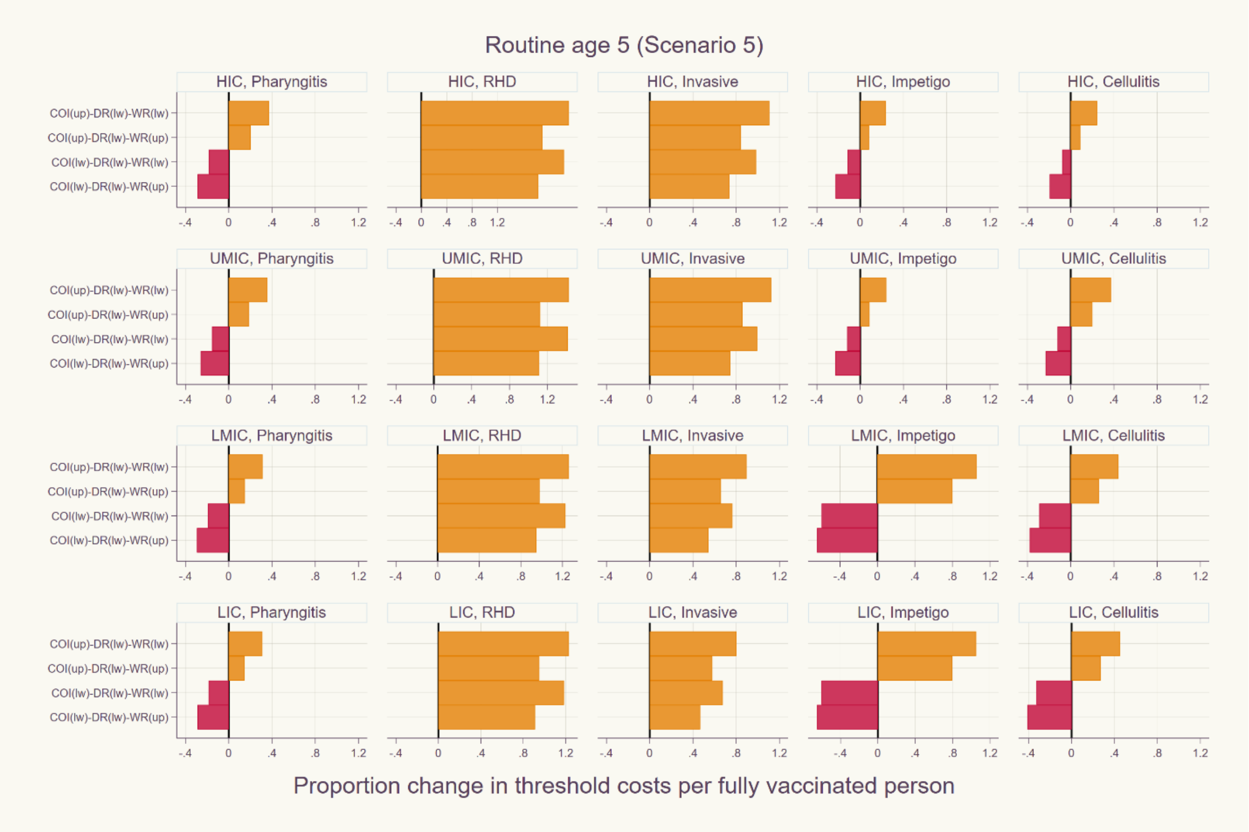
Routine age 5 (Scenario 5)
HIC, Pharyngitis
HIC, RHD
HIC, Invasive
HIC, Impetigo
HIC, Cellulitis
UMIC, Pharyngitis
UMIC, RHD
UMIC, Invasive
UMIC, Impetigo
UMIC, Cellulitis
LMIC, Pharyngitis
LMIC, RHD
LMIC, Invasive
LMIC, Impetigo
LMIC, Cellulitis
LIC, Pharyngitis
LIC, RHD
LIC, Invasive
LIC, Impetigo
LIC, Cellulitis
COI(up)-DR(lw)-WR(lw)
COI(up)-DR(lw)-WR(up)
COI(lw)-DR(lw)-WR(lw)
COI(lw)-DR(lw)-WR(up)
-.4
0
.4
.8
1.2
Proportion change in threshold costs per fully vaccinated person

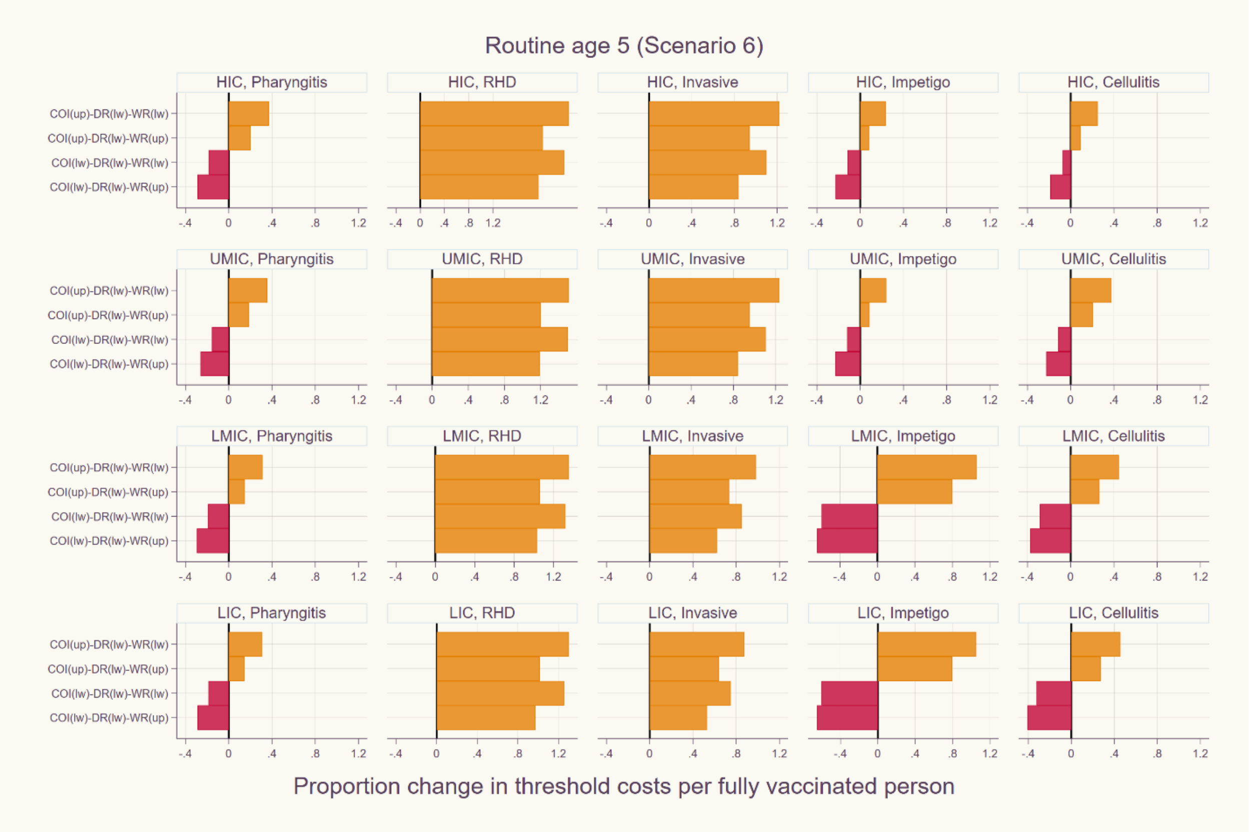
Routine age 5 (Scenario 6)
HIC, Pharyngitis
HIC, RHD
HIC, Invasive
HIC, Impetigo
HIC, Cellulitis
UMIC, Pharyngitis
UMIC, RHD
UMIC, Invasive
UMIC, Impetigo
UMIC, Cellulitis
LMIC, Pharyngitis
LMIC, RHD
LMIC, Invasive
LMIC, Impetigo
LMIC, Cellulitis
LIC, Pharyngitis
LIC, RHD
LIC, Invasive
LIC, Impetigo
LIC, Cellulitis
COI(up)-DR(lw)-WR(lw)
COI(up)-DR(lw)-WR(up)
COI(lw)-DR(lw)-WR(lw)
COI(lw)-DR(lw)-WR(up)
-.4
0
.4
.8
1.2
Proportion change in threshold costs per fully vaccinated person
